# Regional and Sectorial Distribution of Cardiovascular Risk Factors Among Sub-Saharan Africa Workforce: A Systematic Review

**DOI:** 10.7759/cureus.76831

**Published:** 2025-01-02

**Authors:** Abiodun Bamidele Adelowo, Nestor Lemos Ferreira, George Besis, Animesh Gupta, Gideon Mlawa, Zahid Khan

**Affiliations:** 1 Cardiology/Preventative Cardiovascular Medicine, University of South Wales, Wales, GBR; 2 Cardiology, University of South Wales, Wales, GBR; 3 Cardiology, Royal Free Hospital, London, GBR; 4 Acute Internal Medicine, Southend University Hospital NHS Trust, Southend on Sea, GBR; 5 Acute Internal Medicine/Intensive care, Barking, Havering and Redbridge University Hospitals NHS Trust, London, GBR; 6 Internal Medicine and Diabetes and Endocrinology, Barking, Havering and Redbridge University Hospitals NHS Trust, London, GBR; 7 Acute Medicine, Mid and South Essex NHS Foundation Trust, Southend on Sea, GBR; 8 Cardiology, Bart’s Heart Centre UK, London, GBR; 9 Cardiology and General Medicine, Barking, Havering and Redbridge University Hospitals NHS Trust, London, GBR

**Keywords:** cardiology research, cardiovascular disease risk factors, corporate workplace, cvh: cardiovascular health, health education and promotion, health promotion practices, health wellness and promotion, lifestyle medicine, sub-saharan africa, workplace wellness program

## Abstract

The sub-Saharan African region is currently experiencing an unprecedented cardiovascular disease (CVD) epidemic, with CVD accounting for the highest mortality among adults in the region. Changing demographic profiles, lifestyle choices, and preferences for corporate work are identified as root causes of the CVD epidemic in sub-Saharan Africa (SSA). The primary objective of this study was to determine the prevalence of CV risk factors among different regions, countries, and work sectors in SSA. The secondary objective is to identify the work sector with the highest cluster or aggregate of CV risk factors in SSA. This systematic review reports the prevalence of CV risk factors among corporate workers in SSA between 2010 and 2024. Reputable platforms, such as the Cochrane Library, Google Scholar, PubMed, Medline, and Science Direct, were searched for relevant data. A total of 105 studies involving 76,027 participants from nine countries were analyzed. East Africa, Central Africa, West Africa, and Southern Africa had the highest prevalence of unhealthy diet (100%), physical inactivity (80%), high BMI (76%), and metabolic syndrome (MS) (55%), respectively. Ethiopia and South Africa had the highest prevalence of unhealthy diet (100%) and MS (55%), respectively, while Nigeria had the highest prevalence of both stress (71%) and poor sleep (79%). The healthcare work sector had the highest cluster of CV risk factors and the highest prevalence of unhealthy diet (80%), central obesity (51%), and high total cholesterol (36%); the education sector ranked highest in physical inactivity (75%); the administration sector ranked highest in current tobacco smoking (27%) and dysglycemia (17%); and the finance sector workers had the highest prevalence of stress (62%). The prevalence of most risk factors among the corporate workforce in SSA is high, with significant distribution variation across different regions, countries, and work sectors.

## Introduction and background

Although cardiovascular diseases (CVD) account for the highest mortality rate worldwide [1,2], recent advances in public health and clinical interventions have resulted in a progressive decline in the CVD mortality rate globally [3]. However, sub-Saharan Africa (SSA) still experiences an increase in CVD mortality, accounting for approximately 13 % of all deaths annually in the region [4]. Moreover, the prevalence of CVD has reached an epidemic proportion in SSA [5], thereby contributing to the rising double disease burden in the region [6,7]. Because SSA is already grappling with many developmental indices, such as poor economic growth, political instability, insecurity, poverty, high burden of infectious diseases, and fragile healthcare systems, an additional high burden of CVD might be too fatal for the region to handle [8]. Non-communicable diseases (NCDs) are responsible for 7 out of 10 deaths worldwide and more than 15 million people die prematurely every year from a major NCD between the ages of 30 and 69 years. It is important to highlight that more than two-thirds of these premature deaths occur in low- and middle-income countries.

One of the main factors implicated in the rising prevalence and burden of CVD in SSA is the changing demographic profile [4,9]. The median age in Africa is 18.8 years, and 70% of sub-Saharan Africans are below 30 years of age [10,11]. Thus, the SSA region presently houses the youngest population in the world. This age group constitutes the largest productive workforce in the region. However, the demographic profile of the region is changing rapidly. Several risk factors such as family history of CVD, hazardous amount of alcohol intake, lack of physical activity, smoking, unhealthy diet, and lack of healthcare infrastructure and government programs are major issues increasing this risk in the youth. The SSA region is currently experiencing the fastest-growing number of older adults (60 years and above) worldwide [12]. Approximately 46 million older adults lived in SSA in 2015; this number is progressively increasing and is projected to reach 157 million by 2050 [12]. Since aging is an independent risk factor for CVD and about 80% of CVD deaths are usually noticed from around 65 years of age [13], the rapidly changing demographic profile of an older population in SSA may be contributing to the rising epidemic and burden of CVD in the region. More than 50% of premature deaths in the SSA region occur in individuals between 30 and 70 years old and the resulting disability-adjusted life years (DALYs) resulting from this affect this most productive age group, culminating in serious social and economic consequences for their families and society [14]. Additionally, the average health expenditure is below the minimum recommended 44$ per capita in most countries in the SSA region, and individuals have to pay for their healthcare from their pocket due to the lack of universal health coverage in most countries [14]. A situation that may further increase healthcare financing and distort the already fragile healthcare system in many countries in SSA.

Another contributing factor to the CVD epidemic in SSA is the changing work preferences in the region. The increased literacy level, Western influence, and urban settlement have resulted in an increase in corporate work uptake in the region, and Africa is positioned to house the largest and youngest workforce worldwide by 2035 [15]. The choice of white-collar jobs is not without challenges, especially regarding the health of workers. The modern fast-paced, technology-driven corporate workplace environment is often associated with inflexible work schedules, high job demands, hazardous job exposures, and prolonged sitting [16-19]. A situation that may increase the prevalence of some CV risk factors, such as stress, physical inactivity, substance abuse, and hypertension among the youth-dominated workforce [16,17,19-21]. Aside, young people are often more likely to engage in some unhealthy lifestyle practices, such as frequent eating of fast foods, harmful alcohol use, and sleep deprivation, which are also known as CV risk factors [19-22].

However, no systematic review has comprehensively investigated the distribution pattern of CV risk factors among the corporate workforce in different groups of workers in SSA which comprises a younger workforce that is at increasing risk of premature mortality and morbidity as discussed above. In addition, no study has investigated the work sector with the highest cluster of CV risk factors in SSA. These research gaps may have existed due to many prevailing factors in the political, economic, and healthcare systems in many countries in the region, such as lack of political will, insufficient funds, and policies/programs that focus more attention and resources on secondary preventive care less resources on primary preventive care. If these gaps are not quickly and comprehensively addressed, the epidemic and burden of CVD may continue to rise in SSA, especially among the workforce, and may result in an unprecedented CVD mortality rate, slower or decline in economic growth, and the region may be further impoverished.

Aside from these, the corporate workforce is the intellectuals driving the economy, healthcare system, and many critical sectors of most nations worldwide. A high burden of CVD (such as heart attack and stroke) among the corporate workforce in SSA may affect their physical and mental capacities to provide and implement quality economic, healthcare, and other interventions in the region. Thus, the primary objective of this study was to determine the prevalence of CV risk factors across different regions, countries, and work sectors in SSA, while the secondary objective was to identify the work sector in SSA with the highest cluster of CV risk factors. The study was registered with PROSPERO (registration number: CRD42024618275).

## Review

Methodology

The Preferred Reporting Items for Systematic Reviews and Meta-analyses (PRISMA) checklist and the PICO (Population, Investigation/Intervention, Comparison, and Outcomes) protocol were used to guide the conduct of the study. Reliable search engines, such as PubMed, Scopus, Cochrane Library, Medline, Cochrane Library, Google Scholar, and Science Direct, were used to search for relevant data. Some of the keywords searched included cardiovascular risk factors, corporate workers/employees, civil servants, SSA, unhealthy diet, physical inactivity, exercise, sleep, stress, tobacco use/smoking, alcohol use/abuse, hypertension, obesity, diabetes, dysglycemia, dyslipidemia, metabolic syndrome, West Africa, East Africa, Nigeria, Ghana, South Africa, Rwanda, Ethiopia, prevalence, and burden. The PICO protocol includes only the following elements: “Population” (people working corporate workplace or doing a white-collar job in SSA); “Investigation” (cross-sectional, case-control, and cohort studies, randomized control trials (RCTs), systematic reviews, and meta-analyses); “Comparison” (intervention studies that compared the effects of workplace wellness programs on the CVD risk factors among the corporate workforce); while for the “Outcomes” (the studies on the prevalence or changes in CV risk factors, namely unhealthy diet, physical inactivity, stress, poor sleep, tobacco use, khat chewing, alcohol consumption, overweight, obesity, central obesity, hypertension, dysglycemia, dysglycemia, and metabolic syndrome).

**Figure 1:**
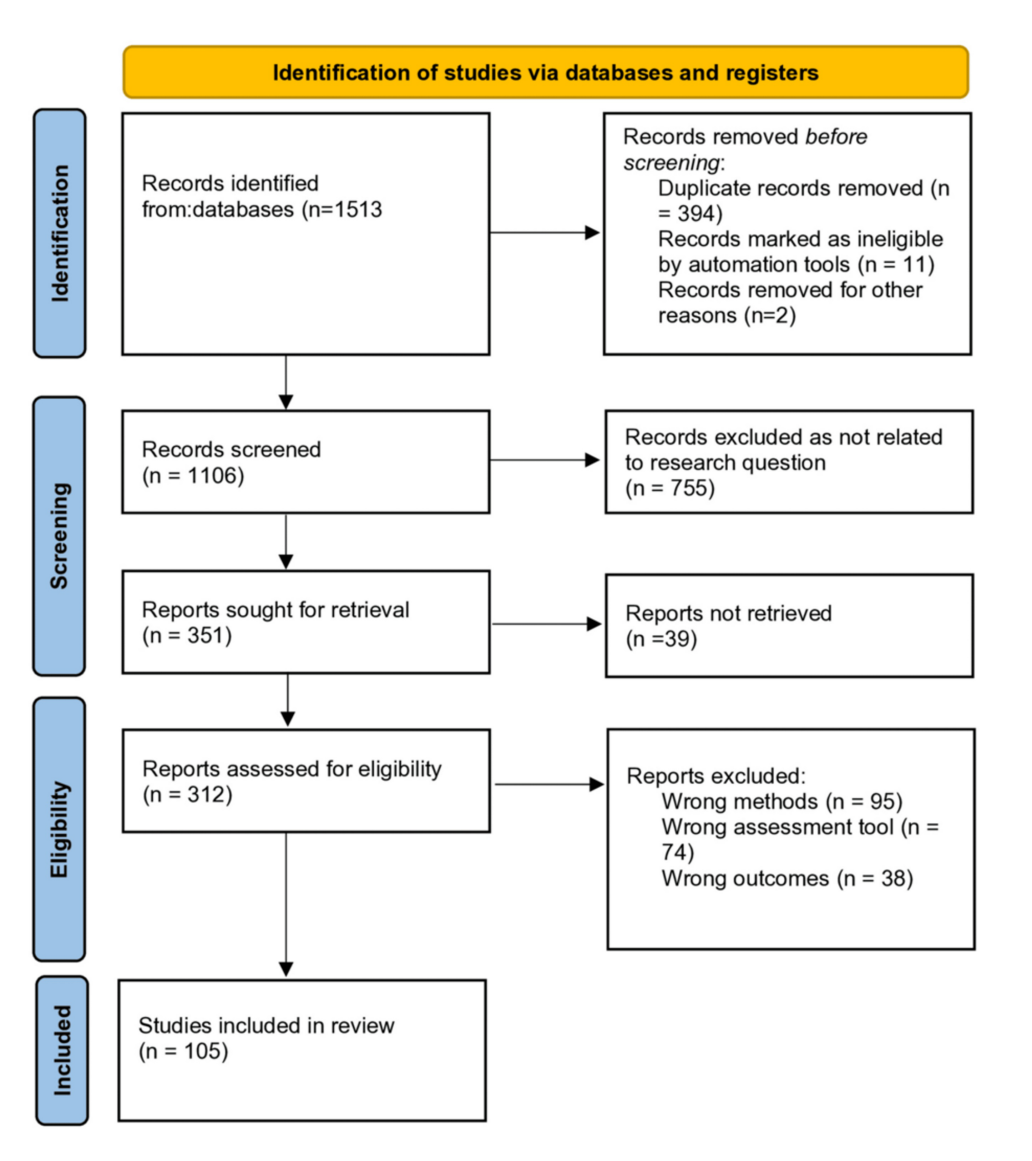
Preferred Reporting Items for Systematic Reviews and Meta-Analyses flow diagram showing the literature The image was created by the authors of this article.

Two independent reviewers performed the search between 1st and 15th October 2024, which identified 1,513 related studies. Subsequently, the Rayyan App was used to remove 394 duplicate articles. Another 755 studies were rejected because they did not meet the inclusion criteria, while 246 studies were screened out for not meeting the other eligibility criteria. Consequently, 105 relevant articles were selected for this study, as shown in the PRISMA chart (Figure 1).

Inclusion criteria

The inclusion criteria were as follows: participants doing a white-collar job or working in a corporate workplace; studies on the prevalence of modifiable CV risk factors (unhealthy diet, physical inactivity, stress, poor sleep, tobacco use, khat chewing, alcohol consumption, overweight, obesity, central obesity, hypertension, dysglyemia, dyslipidemia, and metabolic syndrome); cross-sectional, case-control, and cohort studies; RCTs, systematic reviews, and meta-analyses; public or private corporate workplaces (with more than 10 staff) with a physical location in the SSA; Articles published from January 2010 to March 2024; and articles written in English.

Exclusion criteria

The exclusion criteria were as follows: blue-collar workers (such as miners, construction workers, and truck drivers), full-term athletes, hospital-based studies, case reports, case series, letters to the editor, commentaries, opinion papers, qualitative studies, conference proceedings, and policy papers; studies that were not conducted in SSA; any article published before 2010; and publication in any other language aside from the English language.

Study selection and data extraction

All peer-reviewed studies investigating the prevalence of CV risk factors among corporate workers in SSA were included. Data on the prevalence of the following CV risk factors were extracted from the studies: unhealthy diets, physical inactivity, stress, poor sleep, tobacco use/smoking, khat chewing, alcohol consumption/abuse, overweight/obesity, central obesity, hypertension, dysglycemia, dyslipidemia, and metabolic syndrome.

Data collection process and data items

Data were extracted by two independent reviewers, and Microsoft Excel® was used as a data abstraction form. The form captured the following: title, authors, year of publication, location (country, region, and work sector) of the study, sample size, diagnostic criteria and assessment tools, nature of the intervention, outcomes and other key findings, and limitations of the studies. All variations in the extracted data were discussed and resolved by two reviewers. The data were stored on a personal laptop and the principal investigator's Google Drive account. The Newcastle-Ottawa Scale was used to assess the quality of the included studies (Table 1).

**Table 1:** Modified Newcastle-Ottawa scores for the selected studies Awosan et al. (2013) appeared thrice in the table. The first two (serial numbers 6 and 7) are the same cross-sectional study, but with different work sector study participants. The first group of participants were from the ‘Education’ work sector, while the second group participants were from the ‘finance’ work sector. In the study, the prevalence of the CV risk factors of the study participants from both sectors were assessed and reported separately. The third study (serial number 100) was a separate study conducted in the same year by the researchers.

	Authors	Year	Modified Newcastle-Ottawa scores			
			Selection	Comparability	Outcome	Total
1	Onyango et al. 2017 [23]	2017	4	N/A	3	7
2	Zeleke et al. 2023 [24]	2018	3	N/A	3	6
3	Joshua and Kay 2014 [25]	2014	3		3	6
4	Ambakederemo and Chikezie 2018 [26]	2018	3	N/A	3	6
5	Richard et al. 2016 [27]	2016	4	N/A	3	7
6	Awosan et al. 2013 [28]	2013	4	N/A	3	7
7	Awosan et al. 2013 [28]	2013	4	N/A	3	7
8	Ajewole et al. 2017 [29]	2017	4	N/A	3	7
9	Hope 2023 [30]	2023	3	N/A	3	6
10	Dele-Ojo et al. 2021 [31]	2021	4	N/A	3	7
11	Shitu and Kassie 2021 [32]	2021	4	N/A	3	7
12	Haastrup et al. 2018 [18]	2018	4	N/A	3	7
13	Osei-Yeboah et al. 2018 [33]	2018	4	N/A	3	7
14	Travill et al. 2020 [34]	2019	3	N/A	3	6
15	Jingi and Noubiap 2015 [35]	2015	4	N/A	3	7
16	Olaniyan et al. 2020 [36]	2020	4	N/A	3	7
17	Uwanuruochi et al. 2013 [37]	2013	3	N/A	3	6
18	Olaitan et al. 2020 [38]	2020	4	N/A	3	7
19	Adamu and Abdullahi 2017 [39]	2017	4	N/A	3	7
20	Gyang et al. 2018 [40]	2018	4	N/A	3	7
21	Alao et al. 2022 [41]	2022	4	N/A	3	7
22	Amougou et al. 2019 [42]	2019	4	N/A	3	7
23	Justice et al. 2024 [43]	2021	3	N/A	3	6
24	Odunaiya et al. 2020 [44]	2020	4	N/A	3	7
25	Bernard Ubom et al. 2023 [45]	2023	4	N/A	3	7
26	Khaild et al. 2022 [46]	2022	4	N/A	3	7
27	Adelowo and Sekoni 2013 [47]	2013	4	N/A	3	7
28	Akintunde et al. 2015 [48]	2015	3	N/A	3	6
29	Chukwuemeka et al. 2023 [49]	2023	4	N/A	3	7
30	Salaudeen et al. 2014 [50]	2014	3	N/A	3	6
31	Dele-Ojo et al. 2021 [51]	2021	3	N/A	3	6
32	Chinedu-Eleonu et al. 2021 [52]	2021	3	N/A	3	6
33	Buremoh et al. 2020 [53]	2020	4	N/A	3	7
34	Gebremariam et al. 2018 [54]	2018	3	N/A	3	6
35	Skaal and Pengpid, 2011 [55]	2011	2	N/A	3	5
36	Iwuala et al. 2015 [56]	2015	4	N/A	3	7
37	Onowhakpor et al. 2018 [57]	2018	4	N/A	3	7
38	Ndejjo et al. 2015 [58]	2015	3	N/A	3	6
39	Atuahene et al. 2017 [59]	2017	4	N/A	3	7
40	Ajike and Ezeakunne 2020 [60]	2020	4	N/A	3	7
41	Muluvhu 2018 [61]	2018	4	N/A	3	7
42	Omosivie and Chibianotu 2020 [62]	2020	4	N/A	3	7
43	Sekoni et al. 2013 [63]	2013	4	N/A	3	7
44	Fadeyi et al. 2018 [64]	2018	3	N/A	3	6
45	Monakali et al. 2018 [65]	2018	3	N/A	3	6
46	Aladeniyi et al. 2017 [66]	2017	4	N/A	3	7
47	Hailu et al. 2023 [67]	2023	4	N/A	3	7
48	Diwe et al. 2015 [68]	2015	4	N/A	3	7
49	Adeolu et al. 2016 [69]	2016	4	N/A	3	7
50	Bappah et al. 2022 [70]	2022	4	N/A	3	7
51	Obiebi et al. 2020 [71]	2020	2	N/A	3	5
52	Yakubu and Bigelow 2019 [72]	2019	4	N/A	3	7
53	Mwagi 2018 [73]	2018	4	N/A	3	7
54	Obarisiagbon et al. 2018 [74]	2018	4	N/A	3	7
55	Badego et al. 2020 [75]	2020	4	N/A	3	7
56	Agyemang-Pambour et al. 2023 [76]	2023	4	N/A	3	7
57	Eze and Okorie 2024 [77]	2024	3	N/A	3	6
58	Okwor et al. 2020 [78]	2020	4	N/A	3	7
59	Adeyanju et al. 2023 [79]	2023	3	N/A	3	6
60	Oladimeji et al. 2014 [80]	2014	3	N/A	3	6
61	Paquissi et al. 2016 [81]	2016	2	N/A	3	5
62	Awunor and Isah 2014 [82]	2015	2	N/A	3	5
63	Alinaitwe et al. 2024 [83]	2024	4	N/A	3	7
64	Vincent-Onabajo et al. 2016 [84]	2016	3	N/A	3	6
65	Sumaila et al. 2016 [85]	2016	2	N/A	3	5
66	Akintunde et al. 2014 [86]	2014	3	N/A	3	6
67	Adaja and Idemudia 2018 [87]	2018	3	N/A	3	6
68	Addo et al. 2015 [88]	2015	4	N/A	3	7
69	Egbi et al. 2015 [89]	2015	4	N/A	3	7
70	Angaw et al. 2015 [90]	2015	3	N/A	3	6
71	Olagunju et al. 2021 [91]	2021	3	N/A	3	6
72	Oyeyemi and Adeyemi 2013 [92]	2013	3	N/A	3	6
73	Muluvhu et al. 2020 [93]	2020	3	N/A	3	6
74	Muluvhu et al. 2019 [94]	2019	3	N/A	3	6
75	Adebayo et al. 2020 [95]	2020	3	N/A	3	6
76	Hailu et al. 2022 [96]	2022	3	N/A	3	6
77	Segon et al. 2022 [97]	2022	4	N/A	3	7
78	Kolo et al. 2017 [98]	2017	4	N/A	3	7
79	Olubiyi et al. 2022 [99]	2022	3	0	3	6
80	Burger et al. 2016 [100]	2016	4	N/A	3	7
81	Grace and Semple 2012 [101]	2012	3	N/A	3	6
82	Idris 2019 [102]	2019	3	N/A	3	6
83	Olawuyi and Adeoye 2018 [103]	2018	3	N/A	3	6
84	Enikuomehin et al. 2021 [104]	2021	3	N/A	3	6
85	Etim et al. 2018 [105]	2018	3	N/A	3	6
86	Owolabi et al. 2012 [106]	2012	2	N/A	3	5
87	Capingana et al. 2013 [107]	2013	4	N/A	3	7
88	Mekonen et al. 2022 [108]	2020	3	N/A	3	6
89	Aliyu et al. 2017 [109]	2017	4	N/A	3	7
90	Agyei et al. 2019 [110]	2019	3	N/A	3	6
91	Agyei et al. 2019 [110]	2019	3	N/A	3	6
92	Olatona et al. 2014 [111]	2014	4	N/A	3	7
93	Onyishi et al. 2022 [112]	2022	3	N/A	3	6
94	Chukwu 2023 [113]	2023	3	N/A	3	6
95	Makinde and Salawu 2021 [114]	2021	3	N/A	3	6
96	Ogba 2020 [115]	2020	3	N/A	3	6
97	Sime et al. 2022 [116]	2022	4	N/A	3	7
98	Bosu 2016 [117]	2016	4	N/A	3	7
99	Agbana et al. 2017 [118]	2017	3	2	3	8
100	Awosan et al. 2013 [119]	2013	3	2	3	8
101	Schouw et al. 2020 [120]	2020	3	2	3	8
102	Abiodun 2021 [121]	2021	3	2	3	8
103	Adelowo et al. 2020 [122]	2020	3	2	3	8
104	Edries et al. 2013 [123]	2013	4	2	3	9
105	Torres et al. 2020 [124]	2020	3	2	3	8

Statistical analysis

The Review Manager (RevMan) 5.4 was used for data analysis. The overall prevalence of each CV risk factor was determined by analyzing the sample sizes and prevalence of data across at least three studies. Summary statistics were expressed as percentages with 95% confidence intervals (CIs). Cochran’s Q test was used to assess the statistical heterogeneity in effect sizes. A significant Q-value indicated the presence of heterogeneity. The studies were distributed across regions and sectors based on the specific countries in SSA and where they were conducted and the type (nature) of work of the workforce, respectively.

Furthermore, the prevalence of the total variance attributed to study heterogeneity was calculated using I2 statistics. The degree of heterogeneity was categorized as low, moderate, or high based on I2 values <25%, 25-75%, and > 75%, respectively. This method of categorizing heterogeneity was chosen because it is easy to apply and interpret for multiple variables (CV risk factors) of the study. The statistical significance threshold was set at P < 0.05. Egger’s test was performed to examine the potential presence of publication bias and funnel plots were generated. This ensures effective visual assessment of the distribution of study effects.. Additionally, a leave-one-out sensitivity test was conducted to evaluate the robustness of the findings and to determine the influence of individual studies on the pooled estimates. This process involved systematically excluding each study from the analysis and recalculating the pooled estimates to evaluate their impact on the overall results.

Results

**Figure 2:**
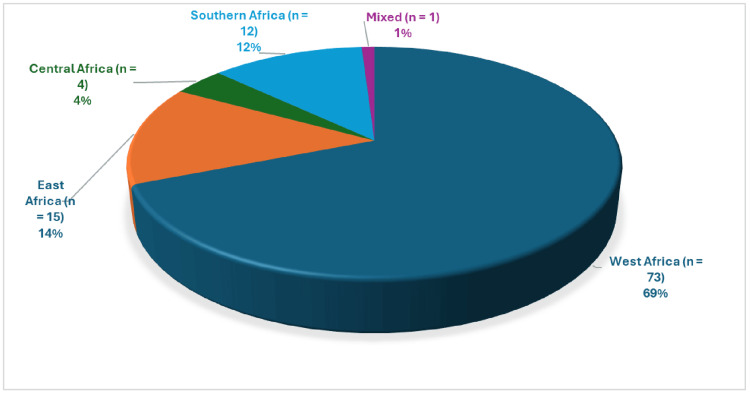
Distribution of studies from the sub-Sahara African regions included in this review The image was created by the authors of this article.

**Figure 3:**
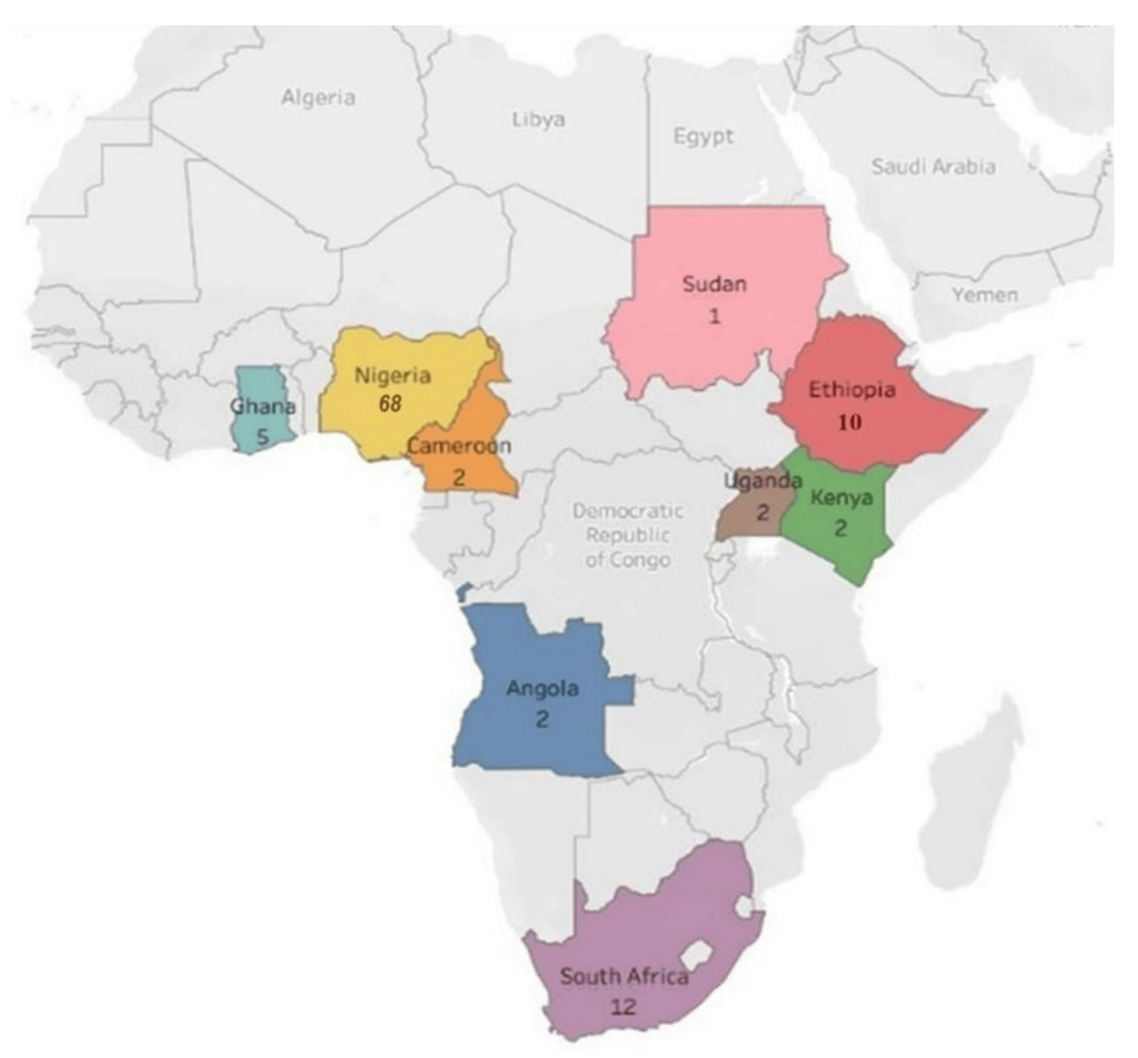
The Sub-Saharan Africa country-wise distribution of studies included in this systematic review This image was created by the authors of this article.

**Figure 4:**
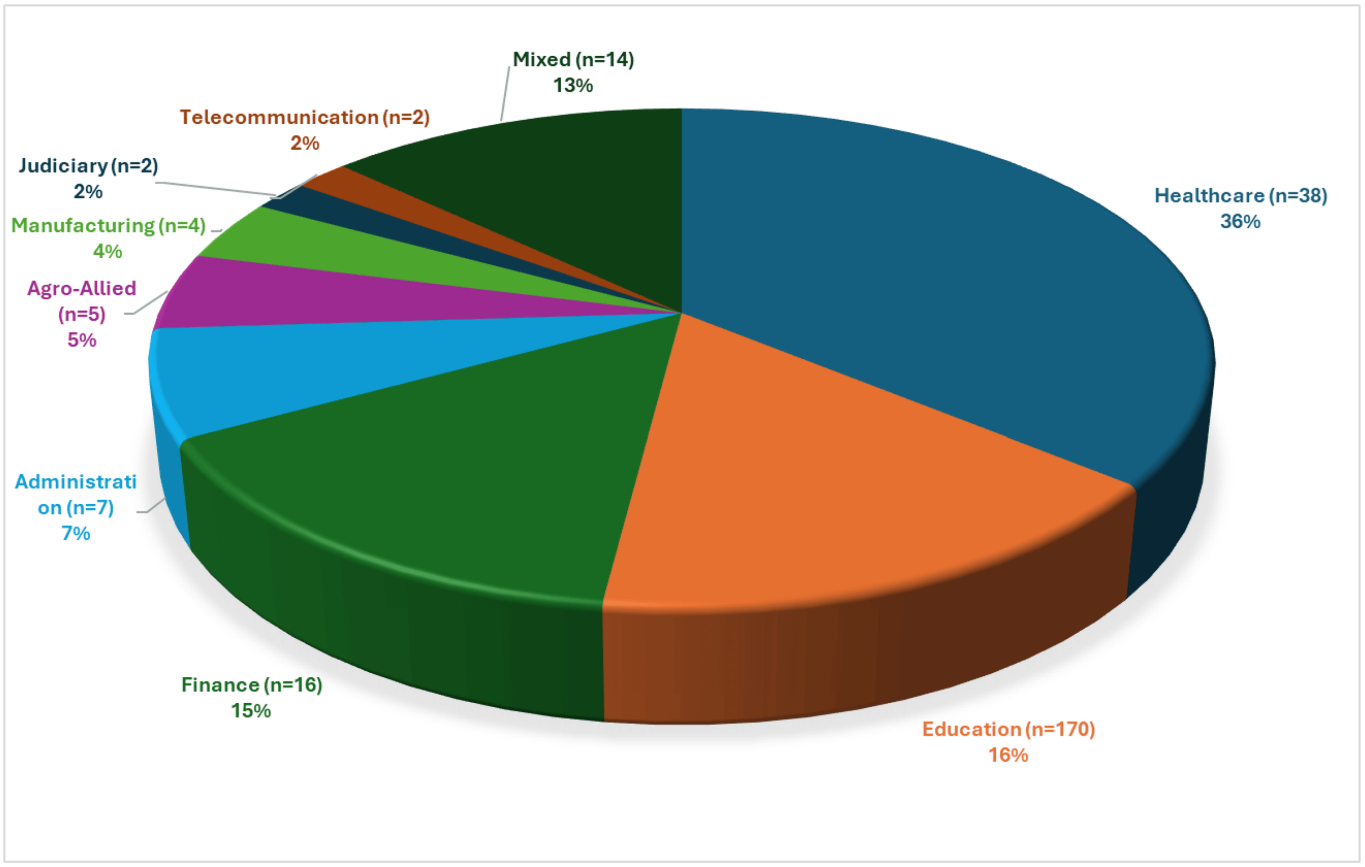
The distribution of included studies based on work sectors The image was created by the authors of this article.

This study included 105 studies and 76,027 respondents. Of these, 97 studies were cross-sectional (18,23-116), one each was a systematic review (117), case-control studies (118), quasi-experimental studies (119), and five RCTs (120-124) (Table 2). Geographically, the majority of 73 (69%) of the studies were conducted in West Africa, followed by 15 (14%) in East Africa, then the South African region with 12 (12%) (Figure 2). The majority (68) of the studies were conducted in Nigeria, followed by South Africa (12), Ethiopia (10), Ghana (5), Cameroon (2), Angola (2), Kenya (2), Uganda (2), and Sudan (10). The countries were identified based on the included studies that were conducted in each country (Figure 3). The studies were conducted across eight distinct work sectors: administration, 7 (7%); agro-allied, 5 (5%); education, 17 (16%); finance, 16 (15%); healthcare, 38 (36%); judiciary, 2 (2%); manufacturing, 4 (4%); and telecommunication, 2 (2%). Although a few studies included participants from different corporate work sections, 14 (13 %) were from a mixed sector (Figure 4).

**Table 2:** Study population demographic characteristics RCT: Randomized controlled trial; CV: cardiovascular; HF: heart failure; MACE: major adverse cardiovascular events; HHF: hospitalization for HF;T2DM: type 2 diabetes mellitus; CKD: chronic kidney disease; PI: physical inactivity; SD: standard deviation; KCCQ-CS: Kansas City Cardiomyopathy Questionnaire- Clinical Summary Score; KCCQ-OS: Kansas City Cardiomyopathy Questionnaire overall summary; KCCQ-PL: Kansas City Cardiomyopathy Questionnaire-physical limitation; KCCQ-TSS: Kansas City Cardiomyopathy Questionnaire-Total symptoms; HFpEF: heart failure with preserved ejection fraction; HFrEF: heart failure with reduced ejection fraction; LVEDV: left ventricular end diastolic volume; LVESV: left ventricular end systolic volume; QoL: quality of life; EEA: European Economic Area; MI: myocardial infarction; ASCVD: atherosclerotic cardiovascular disease; CKD: chronic kidney disease; CREDENCE*: canagliflozin and renal endpoints in diabetes with established nephropathy clinical evaluation; DAPA-CKD: dapagliflozin in patients with kidney disease, with and without heart failure; DELIVER: dapagliflozin evaluation to improve the lives of patients with preserved ejection fraction heart failure; EMBRACE-HF: empagliflozin effects on pulmonary artery pressure in patients with heart failure; EMPA-TROPISM: empagliflozin in nondiabetic patients with heart failure and reduced ejection fraction; EMPEROR-preserved: empagliflozin outcome trial in patients with chronic heart failure with preserved ejection fraction; EMPULSE: empagliflozin in patients hospitalized for acute heart failure; PRESERVED-HF: effects of dapagliflozin on biomarkers, symptoms and functional status in patients with preserved ejection fraction heart failure; VERTIS CV: eValuation of ertugliflozin efficacy and safety cardiovascular outcomes; QoL: quality of life.

Authors	Location (Region)	Sector of Work	Study Design	Sample Size	Diagnostic Criteria/ Assessment Tools	Outcomes
Onyango et al. 2017 [23]	Kenya (East Africa)	Telecommunication	Descriptive cross-sectional	370	Hypertension = ≥140/90mmHg or history of hypertension	Hypertension = 30%
						Overweight = 49.5%
						Obesity = 25.1%
Zeleke et al. 2023 [24]	Ethiopia (East Africa)	Healthcare	Descriptive cross-sectional	450	Hypertension = ≥140/90mmHg or history of hypertension	Current tobacco smoking = 4.2%
						Current alcohol drinking = 66.7%
					Unhealthy diet = < 5 servings of fruits & vegetables/day	Physical inactivity = 4.8%
						Unhealthy diets = 99.6%
						Overweight = 37.1%
						Obesity = 6.9%
					Total cholesterol (TC) = ≥ 200mg/dl	Central obesity = 80.2%
						Hypertension = 23.6%
					LDL-cholesterol (LDL-c) = >130mg/dl	High TC = 28.2%
						High LDL = 25.1%
					Triglycerides (TG) = ≥ 150mg/dl	High TG = 19.3%
						Low HDL-c = 41.3%
						Dyslipidemia = 49.6%
						Dysglycemia = 2.4%
Joshua and Kay 2014 [25]	Nigeria (West Africa)	Education	Descriptive cross-sectional	600	Exercise stage assessment scale	Physical inactivity = 59.6%
Ambakederemo and Chikezie 2018 [26]	Nigeria (West Africa)	Healthcare	Descriptive cross-sectional	169	WHO STEPwise approach guidelines	Overweight = 39.1%
						Obesity = 13.6%
						Central obesity = 37.3%
						Hypertension = 22.1%
						Current smokers = 5.3%
						Current alcohol intake = 36.1%
						Physical inactivity = 74.6%
						No fruits intake/day = 74.6%
						Extra salt intake = 8.9%
						Unhealthy diet = 79.2%
Richard et al. 2016 [27]	Nigeria (West Africa)	Agro-Allied	Descriptive cross-sectional	510	WHO STEPwise approach guidelines	Current Tobacco smokers = 6.9%
					Hypertension = ≥ 140/90 mmHg	Harmful alcohol intake = 15.1%
						Unhealthy diet = 89.8%
						Physical inactivity = 66.5%
						Overweight = 13.9%
						Obesity = 3.9%
						Hypertension = 37.1%
Awosan et al. 2013 [28]	Nigeria (West Africa)	Education	Descriptive cross-sectional	110	WHO STEPwise approach guidelines	Unhealthy diet = 61.9%
						Physical inactivity = 5.7%
						Current alcohol intake = 3.8%
						Current tobacco smokers = 4.80%
						Stress = 52.4%
						Overweight = 22.9%
						Obesity = 26.7%
						Hypertension = 33.4%
						Diabetes = 10.5%
						High TC = 37.1%
Awosan, KJ, et al., 2013 [28]	Nigeria (West Africa)	Finance	Descriptive cross-sectional	110	WHO STEPwise approach guidelines	Physical inactivity = 33.3%
						Overweight = 45.7%
						Obesity = 19.0%
						Current Tobacco smokers = 7.6%
						Hypertension = 22.9%
						Diabetes = 8.60%
						Unhealthy diet = 77.1%
						Alcohol intake = 27.6%
						High TC = 41.9%
						Stress = 43.8%
Ajewole et al. 2017 [29]	Nigeria (West Africa)	Education	Descriptive cross-sectional	203	Hypertension = ≥ 140/90 mmHg	Hypertension = 5.8%
						Alcohol intake = 40.9%
						Unhealthy diet = 7.9%
						Current tobacco smokers = 9.4%
Hope 2023 [30]	Nigeria (West Africa)	Healthcare	Descriptive cross-sectional	140	Hypertension = JNC VII criteria	Hypertension = 32.1%
						Overweight = 53.6%
						Obesity = 23.6%
						Diabetes = 17.9%
						Alcohol intake = 27.9%
						Current tobacco smokers = 9.3%
						Physical inactivity = 80.0%
						Unhealthy diet = 20.0%
Dele-Ojo et al. 2021 [31]	Nigeria (West Africa)	Healthcare	Descriptive cross-sectional	248	Hypertension = ≥140/90mmHg	Obesity = 28.6%
						Central obesity = 60.9%
						Hypertension = 38.7%
						Dysglycemia = 35.1%
						High TC = 50.4%
						High LDL-c = 37.9%
						Low HDL-c = 14.5%
						High TG = 24.2%
Shitu and Kassie 2021 [32]	Ethiopia (East Africa)	Finance	Descriptive cross-sectional	368	WHO STEPwise approach guidelines	Hypertension = 52.4%
						Overweight = 28.0%
					General Physical Activity Questionnaire (GPAQ)	Obesity = 15.0%
						No vegetables/day = 15.2%
					AUDIT	No fruits/day = 6.5%
						High salt intake = 43.5%
						Current alcohol intake = 76.1%
						Current tobacco smokers = 4.4%
						Physical inactivity = 50.3%
						Stress = 65.2%
Haastrup et al. 2018 [18]	Nigeria (West Africa)	Administration	Descriptive cross-sectional	184	WHO STEPwise approach guidelines	Unhealthy diet = 90.8%
						Extra salt intake = 42.9%
						Current Tobacco smokers = 23.4%
						Passive Tobacco smokers = 18.4%
						Alcohol intake = 43.5%
						Harmful Alcohol intake = 90.0%
						Physical inactivity = 64.1%
						Overweight = 42.4%
						Obesity = 14.7%
						Central obesity = 48.4%
						Hypertension = 33.7%
						Dysglycemia = 24.0%
						High TC = 28.8%
Osei-Yeboah et al. 2018 [33]	Ghana (West Africa)	Healthcare	Descriptive cross-sectional	112	Hypertension = JNC VII criteria	Hypertension = 16.1%
						Overweight = 38.39%
						Obesity = 12.50%
						Dysglycemia = 9.92%
						Dyslipidemia = 26.79%
						High TC = 18.75%
						High TG = 10.71%
Travill et al. 2020 [34]	South Africa (Southern Africa)	Manufacturing	Descriptive cross-sectional	75	American College of Sports Medicine’s guidelines	Overweight = 32%
						Obesity = 32%
						Hypertension = 55%
						Dysglycemia = 25.3%
						High TC = 28%
Jingi and Noubiap 2015 [35]	Cameroon (Central Africa)	Healthcare	Descriptive cross-sectional	65	Hypertension = JNC VII criteria	Hypertension = 26.2%
					WHO STEPwise approach guidelines	Diabetes = 3.1%
						Overweight = 46.2%
						Obesity = 23.1%
						Current tobacco smokers = 12.3%
						Harmful alcohol intake = 61.5%
						Physical inactivity = 16.9%
Olaniyan et al. 2020 [36]	Nigeria (West Africa)	Mixed	Descriptive cross-sectional	260	Hypertension = JNC VII criteria	Hypertension = 40.4%
					WHO STEPwise approach guidelines	Obesity = 52.3%
						Central obesity = 35.0%
						Current tobacco smokers = 5.8%
						Alcohol intake = 26.5%
						Physical inactivity = 75.8%
						Diabetes = 38.1%
						Lipid Profile:
						High TC = 55.4%
						High LDL-c = 85.0%
						High TG = 3.1%
Uwanuruochi et al. 2013 [37]	Nigeria (West Africa)	Healthcare	Descriptive cross-sectional	299	Hypertension = WHO/ISH criteria	Hypertension = 37.5%
						Obesity = 42.1%
						Dysglycemia = 20.8%
						High TC = 18.1%
					WHO STEPwise approach guidelines	High LDL-c = 26.8%
						Low HDL-c = 41.9%
						High TG = 9.7%
						Metabolic syndrome = 24.7%
Olaitan et al. 2020 [38]	Nigeria (West Africa)	Healthcare	Descriptive cross-sectional	283	JNC VII criteria	Alcohol intake = 21.6%
						Current tobacco smoking = 2.5%
						Stress = 60.9%
						Physical inactivity = 55.1%
					International Stress Management Association Questionnaire (ISMAQ)	Diabetes = 1.4%
						Central obesity = 47.3%
						High BMI = 49.5%
						Hypertension = 30.1%
Adamu and Abdullahi 2017 [39]	Nigeria (West Africa)	Healthcare	Descriptive cross-sectional	196	Perceived stress screening tool	Stress = 55.9%
Gyang et al. 2018 [40]	Nigeria (West Africa)	Healthcare	Descriptive cross-sectional	155	Hypertension = >140/>90mmHg	Hypertension = 41.9%
						Physical inactivity = 92.4%
						High BMI = 49.6%
					Poor sleep = < 8 Hours/day	Central obesity = 58.8%
						Dysglycemia = 45.8%
						Poor Sleep = 48.9%
Alao et al. 2022 [41]	Nigeria (West Africa)	Healthcare	Descriptive cross-sectional	232	Stress = Work-Related Quality of Life (WRQoL) scale	Stress = 62.1%
Amougou et al. 2019 [42]	Cameroon (Central Africa)	Healthcare	Descriptive cross-sectional	350	WHO STEPwise approach guidelines	Obesity = 30.3%
						Central obesity = 46.9%
						Hypertension = 6.3%
					Unhealthy diet = < 400 g of fruits and vegetables/day or < 5 portions of fruits and vegetables/day	Diabetes = 2.9%
						Current tobacco smokers = 2.0%
						Unhealthy diet = 99.1%
Justice et al. 2024 [43]	Ghana (West Africa)	Finance	Descriptive cross-sectional	136	BMI: Weight/(Height)^2^	Overweight = 31.6%
						Obesity = 30.9%
Odunaiya et al. 2020 [44]	Nigeria (West Africa)	Healthcare	Descriptive cross-sectional	316	International Physical Activity Questionnaire (IPAQ)	Hypertension = 6.0%
					AUDIT-C	Overweight = 34.1%
						Obesity = 42.6%
						Physical inactivity = 49.1%
						Alcohol intake = 0.95%
Bernard Ubom et al. 2023 [45]	Nigeria (West Africa)	Healthcare	Descriptive cross-sectional	629	Validated self-developed tool using WHO guideline	Physical inactivity = 73.1%
						Diabetes = 0.8%
						Current smoking = 3.9%
						Alcohol intake = 22.5%
Khaild et al. 2022 [46]	Sudan (East Africa)	Finance	Descriptive cross-sectional	98	High salt = Extra salt to meals	Hypertension = 45.9%
					Unhealthy diet = Fruits intake < 3 times/week	High BMI = 40.8%
						Current Tobacco smokers = 20%
						High salt intake = 53.1%
						Unhealthy diet = 50%
						Physical inactivity = 38.8%
						Stress = 96%
Adelowo and Sekoni 2013 [47]	Nigeria (West Africa)	Finance	Descriptive cross-sectional	260	WHO STEPwise guidelines	Unhealthy diet = 7.3%
						High salt intake = 19.3%
						Current tobacco smokers = 10%
					Unhealthy diet = < 5 servings of fruits and/or vegetables/day	Alcohol intake = 42.7%
						Harmful alcohol intake = 27.1%
						Physical inactivity = 44.2%
Akintunde et al. 2015 [48]	Nigeria (West Africa)	Education	Descriptive cross-sectional	206	Heart Disease Fact Questionnaire (HDFQ)	Obesity = 38.3%
Chukwuemeka et al. 2023 [49]	Nigeria (West Africa)	Education	Descriptive cross-sectional	70	Cardiovascular disease risk factors knowledge level (CARRF-KL) questionnaire	Hypertension = 12.9%
						Stress = 48.6%
						Overweight = 51.4%
					Cardiovascular risk factor (CRF) questionnaire	Obesity = 11.4%
					ISMAQ	Central obesity = 21.4%
						Physical inactivity = 18.6%
					IPAQ-SF	Dysglycemia = 7.1%
						Current smokers = 7.1%
						Alcohol intake = 20.0%
Salaudeen et al. 2014 [50]	Nigeria (West Africa)	Finance	Descriptive cross-sectional	180	BMI = Weight/(Height)^2^	Current Tobacco smokers = 32.2%
						Alcohol intake = 41.7%
						Unhealthy diet = 77.8%
						Physical inactivity = 75.0%
						Overweight = 14.4%
						Obesity = 20.0%
Dele-Ojo et al. 2021 [51]	Nigeria (West Africa)	Education	Descriptive cross-sectional	223	Heart Disease Fact Questionnaire (HDFQ)	Hypertension = 35.4%
					Unhealthy diet = < 5 servings of fruits/vegetables daily	Diabetes = 12.1%
						Overweight = 31.8%
						Obesity = 23.3%
					Hypertension = JNC VII criteria	Physical inactivity = 83%
						Unhealthy diet = 67.7%
						Current Tobacco smokers = 2.2%
Chinedu-Eleonu et al. 2021 [52]	Nigeria (West Africa)	Healthcare	Descriptive correlational	388	Hypertension = ≥140/90mmHg	Hypertension = 36.1%
Buremoh et al. 2020 [53]	Nigeria (West Africa)	Healthcare	Descriptive cross-sectional	196	Unhealthy diet = < 5 servings of fruits/vegetables/day	Unhealthy diet = 91.8%
						Physical inactivity = 77%
						Current smokers = 20.9%
						Alcohol intake = 21.9%
					Poor sleep = < 5-6 hours/nights	Poor sleep = 41%
						Central obesity = 58.2%
						Hypertension = 40.8%
						Dysglycemia = 1.5%
Gebremariam et al. 2018 [54]	Ethiopia (East Africa)	Mixed	Descriptive cross-sectional	1380	WHO STEPwise approach guidelines	Unhealthy diet = 99.7%
						Current smoker = 2.2%
						Khat chewing = 1.6%
						Alcohol intake = 18.9%
					Unhealthy diet = < 5 servings of fruits/vegetables daily	Physical inactivity = 41.0%
						Overweight = 26.0%
						Obesity = 4.1%
						Central obesity = 27.2%
					Physical inactivity = < 600 MET-minutes/week	High Systolic Blood Pressure = 10.5%
						High Diastolic Blood Pressure = 14.7%
						Dysglycemia = 19.4%
						Diabetes = 40.9%
						High TC = 25.2%
						High LDL-c = 51.6%
						Low HDL-c = 59.2%
						High TG = 55.7%
Skaal and Pengpid, 2011 [55]	South Africa (Southern Africa)	Healthcare	Descriptive cross-sectional	200	BMI = Weight/(Height)^2^	Diabetes = 10.0%
						Hypertension = 20.0%
						Stress = 32.5%
						Overweight = 26.5%
						Obesity = 47.0%
Iwuala et al. 2015 [56]	Nigeria (West Africa)	Healthcare	Descriptive cross-sectional	300	IPAQ-SF	Overweight = 44.7%
						Obesity = 27.3%
						Central obesity = 49.7%
						Physical inactivity = 79.2%
Onowhakpor et al. 2018 [57]	Nigeria (West Africa)	Healthcare	Descriptive cross-sectional	229	General Health Questionnaire (GHQ-12)	Stress = 50.7%
Ndejjo et al. 2015 [58]	Uganda (East Africa)	Healthcare	Descriptive cross-sectional. Multicenter	200	National Institute of Occupational Safety and Health tool	Alcohol intake = 19.0%
						Physical inactivity = 59.0%
					Poor sleep = <8 hours/day	Poor sleep = 75.0%
						Stress = 21.5%
Atuahene et al. 2017 [59]	Ghana (West Africa)	Mixed	Descriptive cross-sectional	271	BMI = Weight/(Height)^2^	Alcohol intake = 47.9%
						Current tobacco smoker = 2.7%
						Physical inactivity = 63.6%
						Overweight = 29.9%
						Obesity = 4.8%
Ajike and Ezeakunne 2020 [60]	Nigeria (West Africa)	Finance	Descriptive cross-sectional	198	Validated self-developed structured tool	Stress = 94.4%
Muluvhu 2018 [61]	South Africa (Southern Africa)	Administration	Descriptive cross-sectional	535	IPAQ	Dysglycemia = 25%
						Physical inactivity = 77%
						Overweight = 27%
					NCEP-ATPIII criteria	Obesity = 34%
						Central obesity = 64%
						Hypertension = 25%
					IDF diagnostic criteria	Metabolic syndrome = 55%
						Alcohol intake = 29%
						Current tobacco smoker = 51%
Omosivie and Chibianotu 2020 [62]	Nigeria (West Africa)	Judiciary	Descriptive cross-sectional	226	WHO STEPwise approach guidelines	Hypertension = 47.3%
						High BMI = 65.5%
					Unhealthy diet = Fruits/Vegetables intake < twice/week	Current smoker = 8.8%
						Alcohol intake = 28.8%
						Unhealthy diet = 37.6%
						High salt intake = 30.5%
					High salt intake = Adding salt to food before eating or self-grading salt consumption as high	Physical inactivity = 39.8%
						Diabetes = 10.2%
Sekoni et al. 2013 [63]	Nigeria (West Africa)	Finance	Descriptive cross-sectional	260	Hypertension = ≥140/90mmHg	Hypertension = 29.6%
					BMI: Weight/(Height)^2^	High BMI = 40.4%
Fadeyi et al. 2018 [64]	Nigeria (West Africa)	Healthcare	Descriptive cross-sectional	88	Poor sleep = < 7 hours/night	Current smoker = 1.1%
						Alcohol intake = 4.6%
						High BMI = 29.6%
						Poor sleep = 47.7%
Monakali et al. 2018 [65]	South Africa (Southern Africa)	Healthcare	Descriptive cross-sectional	203	Modified WHO STEPwise approach guidelines	Hypertension = 52%
						Alcohol intake = 26.6%
						Current smokers = 8.4%
						Physical inactivity = 32.5%
						Obesity = 46.8%
Aladeniyi et al. 2017 [66]	Nigeria (West Africa)	Mixed	Descriptive cross-sectional	4844	Modified WHO STEPwise approach guidelines	Hypertension = 35%
						Poor sleep = 85.2%
					Poor sleep = < 6 hours/day	Alcohol intake = 8.5%
						Physical inactivity = 60.6%
Hailu et al. 2023 [67]	Ethiopia (East Africa)	Manufacturing	Descriptive cross-sectional	370	Pittsburgh Sleep Quality Index (PSQI)	Overweight = 15.1%
					Food and Agriculture Organization’s Individual Dietary Diversity Score (IDDS)	Obesity = 4.1%
						Physical inactivity = 68.4%
						Current tobacco smokers = 8.6%
						Khat chewing = 4.1%
						Alcohol intake = 52.4%
						Poor sleep = 75.4%
Diwe et al. 2015 [68]	Nigeria (West Africa)	Finance	Descriptive cross-sectional	194	Hypertension = <140/90mmHg	Hypertension = 12.4%
						Alcohol intake = 50%
					BMI = Weight/(Height)^2^	Current Tobacco smokers = 8.3%
						Obesity = 37.5%
Adeolu et al. 2016 [69]	Nigeria (West Africa)	Healthcare	Descriptive cross-sectional	253	GHQ-12	Stress = 31.6%
					General practitioner job stress inventory	
Bappah et al. 2022 [70]	Nigeria (West Africa)	Education	Descriptive cross-sectional	281	GPAQ	Hypertension = 27.8%
						Overweight = 29.9%
						Obesity = 17.4%
						Current tobacco smokers = 12.1%
						Physical inactivity = 47.7%
Obiebi et al. 2020 [71]	Nigeria (West Africa)	Healthcare	Descriptive cross-sectional	232	HBP = ≥140/90mmHg	Hypertension = 36.2%
					BMI = Weight/(Height)^2^	Overweight = 41.4%
						Obesity = 20.3%
						Central obesity = 56.0%
						Physical inactivity = 26.3%
Yakubu and Bigelow 2019 [72]	Nigeria (West Africa)	Finance	Descriptive cross-sectional	3013	HBP = ≥140/90mmHg	Overweight = 39.3%
					BMI = Weight/(Height)^2^	Obesity = 23.6%
						Hypertension = 27.6%
Mwagi 2018 [73]	Kenya (East Africa)	Telecommunication	Descriptive cross-sectional	400	Modified WHO STEPwise approach guidelines	Hypertension = 29.7%
						Poor sleep = 22%
						Overweight = 40.3%
					Unhealthy diet = < 5 servings of fruits/vegetables daily	Obesity = 24.8%
						Unhealthy diet = 66.7%
						High salt intake = 60.4%
					Poor sleep = ≤ 5 hours	Physical inactivity = 87.5%
						Alcohol intake = 55.0%
						Current tobacco smoker = 4.0%
Obarisiagbon et al. 2018 [74]	Nigeria (West Africa)	Agro-allied	Descriptive cross-sectional	354	WHO STEPwise approach guidelines	Hypertension = 18.4%
						Current Tobacco smokers = 4.5%
						Alcohol intake = 48.6%
					Hypertension = ≥140/90 mmHg	Overweight = 25.4%
						Obesity = 9.6%
						Diabetes = 2.8%
Badego et al. 2020 [75]	Ethiopia (East Africa)	Mixed	Descriptive cross-sectional	546	WHO STEPwise approach guidelines	Hypertension = 24.5%
					Unhealthy diet = Insufficient fruit intake ≤ 6 days/week and/or Insufficient vegetable intake ≤ 6 days/week	Diabetes = 5.7%
						Alcohol intake = 16.5%
						Current tobacco smokers = 0.4%
						Khat chewing = 12.6%
						Physical inactivity = 29.5%
						Insufficient fruits intake = 91.2%
						Insufficient vegetable intake = 67.3%
						High salt intake = 9.2%
						Overweight = 42.7%
						Obesity = 17.8%
Agyemang-Pambour et al. 2023 [76]	Ghana (West Africa)	Mixed	Descriptive cross-sectional	173	WHO STEPwise Approach guidelines	Hypertension = 29.3%
					GPAQ	Alcohol intake = 13.5%
					Hypertension = JNC VII criteria	Physical inactivity = 53.5%
						Overweight = 33.5%
						Obesity = 21.4%
Eze and Okorie 2024 [77]	Nigeria (West Africa)	Judiciary	Descriptive cross-sectional	120	Hypertension = ≥140/90 mmHg	Hypertension = 25.8%
					Harmful Alcohol Intake = Intake of > 14 units/ week in men and > 7 units/ week in women	Overweight = 27.5%
						Obesity = 6.7%
						Dysglycemia = 5.8%
						Dyslipidemia = 3.3%
						Harmful alcohol intake = 5.0%
						Current tobacco smokers = 1.7%
Okwor et al. 2020 [78]	Nigeria (West Africa)	Finance	Descriptive cross-sectional	370	Stress = Health, Safety, Executive Management Standards Indicator Tool (HSE-MS IT)	Stress = 47%
Adeyanju et al. 2023 [79]	Nigeria (West Africa)	Mixed	Descriptive cross-sectional	296	Hypertension = ≥140/90mmHg	Hypertension = 33.4%
						Overweight = 43.6%
						Obesity = 15.2%
						Alcohol intake = 48.0%
						Current tobacco smokers = 7.4%
						Physical inactivity = 58.1%
Oladimeji et al. 2014 [80]	Nigeria (West Africa)	Mixed	Descriptive cross-sectional	801	Unhealthy diet = No fresh fruits and cooked Vegetables/day	Hypertension = 29%
					PI = < 30 minutes vigorous physical activity < 5 days/week	Overweight = 35%
					Harmful alcohol intake = ≥5 alcohol drinks intake at one sitting in men and ≥4 drinks in females	Obesity = 27%
						Physical inactivity = 91%
						Unhealthy diet = 90%
						Current smokers = 6%
						Harmful Alcohol intake = 2%
Paquissi et al. 2016 [81]	Angola (Central Africa)	Education	Descriptive cross-sectional	781	Hypertension = JNC VII guideline	Hypertension = 17.9%
						Dysglycemia = 10.6%
						Overweight = 34.4%
						Obesity = 19.9%
						Current smoking = 4.9%
						Harmful Alcohol intake = 45.3%
Awunor and Isah 2014 [82]	Nigeria (West Africa)	Agro-Allied	Descriptive cross-sectional	349	Hypertension = WHO-ISH criteria	Hypertension = 28.1%
						Diabetes = 1.4%
						Obesity = 8.3%
						Alcohol intake = 49.0%
						Current smokers = 5.4%
						Physical inactivity = 43.8%
Alinaitwe et al. 2024 [83]	Uganda (East Africa)	Education	Descriptive cross-sectional	141	Modified WHO STEPwise approach guidelines	Hypertension = 26.2%
						Physical inactivity = 78.7%
						Overweight = 46.8%
						Obesity = 20.6%
						Unhealthy diet = 100%
						High salt intake = 46.8%
						Alcohol intake = 51.1%
Vincent-Onabajo et al. 2016 [84]	Nigeria (West Africa)	Education	Descriptive cross-sectional	441	Hypertension = ≥140/90mmHg	Hypertension = 36.1%
						Overweight = 39.9%
						Obesity = 22.2%
Sumaila et al. 2016 [85]	Nigeria (West Africa)	Healthcare	Descriptive cross-sectional	107	GPAQ	Hypertension = 26.2%
						Physical inactivity = 49.5%
						Unhealthy diet = 29.9%
						Current tobacco smokers = 12.2%
Akintunde et al. 2014 [86]	Nigeria (West Africa)	Education	Descriptive cross-sectional	206	Hypertension = JNC VII criteria	Hypertension = 40.8%
					Dyslipidemia = NCEP panel IV guideline	Obesity = 38.3%
						Central obesity = 44.7%
						Dysglycemia = 9.3%
						High TC = 49.5%
						High LDL-c = 48.1%
						Low HDL-c = 54.9%
Adaja and Idemudia 2018 [87]	Nigeria (West Africa)	Healthcare	Descriptive cross-sectional	325	Dyslipidemia = NCEP panel guideline	Hypertension = 16.0%
					Unhealthy diet = No fruit and vegetable intake/day	Overweight = 31.7%
						Obesity = 25.5%
						Central obesity = 62.2%
						Alcohol intake = 53.5%
						Current tobacco smokers = 3.4%
						Physical inactivity = 68.3%
						Unhealthy diet = 68.9%
						Hight TC = 43.4%
						High LDL-c = 56.0% Low HDL-c = 82.2%
						High TG = 5.5%
Addo et al. 2015 [88]	Ghana (West Africa)	Finance	Descriptive cross-sectional	180	WHO STEPwise approach guidelines	Overweight = 37.8%
						Obesity = 17.8%
					General Practice Physical Activity Questionnaire (GPPAQ)	Alcohol intake = 57.8%
						Physical inactivity = 83.3%
Egbi et al. 2015 [89]	Nigeria (West Africa)	Healthcare	Descriptive cross-sectional	231	Hypertension = ≥140/90 mmHg	Hypertension = 21.3%
						Overweight = 35.5%
						Obesity = 23.8%
						Central obesity = 13.9%
						Alcohol intake = 23.8%
						Current smokers = 2.2%
						Dysglycemia = 2.7%
Angaw et al. 2015 [90]	Ethiopia (East Africa)	Mixed	Descriptive cross-sectional	629	WHO STEPwise approach guidelines	Hypertension = 27.3%
					Unhealthy diet = Fruits and vegetable intake < 4 times/week	Current tobacco smokers = 4.8%
						Khat chewing = 5.0%
						Insufficient fruits intake = 93.6%
						Insufficient vegetable intake = 80.2%
Olagunju et al. 2021 [91]	Nigeria (West Africa)	Healthcare	Descriptive cross-sectional	303	GHQ-12	Stress = 23.4%
					PSQI	Poor sleep = 60.4%
Oyeyemi and Adeyemi 2013 [92]	Nigeria (West Africa)	Mixed	Descriptive cross-sectional	292	IPAQ-SF	History of hypertension = 23.1%
						Metabolic syndrome = 48.8%
						Obesity = 24.0%
						Physical inactivity = 58.5%
Muluvhu et al. 2020 [93]	South Africa (Southern Africa)	Mixed	Descriptive cross-sectional	468	Physical Activity Index (PAI) questionnaire	Hypertension = 25%
						Overweight = 26.0%
						Obesity = 33%
						Alcohol intake = 29%
						Metabolic syndrome = 55%
						Current tobacco smokers = 51%
						Physical inactivity = 77%
Muluvhu et al. 2019 [94]	South Africa (Southern Africa)	Mixed	Descriptive cross-sectional	452	HBP = ≥140/90mmHg	Hypertension = 25%
					BMI = Weight/(Height)^2^	Overweight = 27%
						Obesity = 34%
Adebayo et al. 2020 [95]	Nigeria (West Africa)	Education	Descriptive cross-sectional	847	Hypertension = ≥140/90mmHg	Hypertension = 15.0%
						Overweight = 32%
						Obesity = 25.5%
						Physical inactivity = 86%
					Unhealthy diet = No daily fruit and vegetable intake/day	Current Tobacco smokers = 1.5%
						Alcohol intake = 24%
						No fruit intake/day = 75.5%
						No vegetable intake/day = 81%
Hailu et al. 2022 [96]	Ethiopia (East Africa)	Education	Descriptive cross-sectional	607	PSQI	High BMI = 20.6%
					Workplace Stress Scale	Poor sleep = 60.3%
						Current Tobacco smokers = 17.8%
						Khat chewing = 3.1%
						Alcohol intake = 18.5%
						Physical inactivity = 66.7%
						Stress = 45.5%
Segon et al. 2022 [97]	Ethiopia (East Africa)	Healthcare	Descriptive cross-sectional	510	PSQI	Poor sleep = 75.5%
					DASS-21	Stress = 33.1%
					ASSIST questionnaire	Current Tobacco smokers = 8.4%
						Khat chewing = 9.6%
						Alcohol intake = 33.1%
Kolo et al. 2017 [98]	Nigeria (West Africa)	Healthcare	Descriptive cross-sectional	160	PSQI	Poor sleep = 54.2%
Olubiyi et al. 2022 [99]	Nigeria (West Africa)	Healthcare	Descriptive cross-sectional	301	WHO STEPwise approach	High TC = 62.8%
						High LDL-c = 26.6%
					Dyslipidemia = NCEP panel guideline	Low HDL-c = 7.3%
						High TG = 10.3%
Burger et al. 2016 [100]	South Africa (Southern Africa)	Agro-allied	Descriptive cross-sectional correlational	118	General health questionnaire (GHQ)	Alcohol intake = 34.7%
						Current tobacco smokers = 33.9%
Grace and Semple [101]	South Africa (Southern Africa)	Administration (Hospitality)	Descriptive cross-sectional	137	Hypertension = ≥140/90 mmHg	Hypertension = 34.3%
Idris 2019 [102]	Nigeria (West Africa)	Finance	Descriptive cross-sectional	52	HBP = ≥140/90 mmHg	Hypertension = 28.8%
					BMI = Weight/(Height)^2^	Overweight = 50%
						Obesity = 15.4%
Olawuyi and Adeoye 2018 [103]	Nigeria (West Africa)	Administration	Descriptive cross-sectional	606	WHO STEPwise approach guidelines	Hypertension = 33.1%
					Unhealthy diet = < 5 servings of fruits and vegetables/day. Harmful alcohol intake = > 5 drinks in men or > 4 drinks in women on one or more occasion within 30 days	High BMI = 57.3%
						Central obesity = 37.1%
						Current Tobacco smokers = 6.5%
					IPAQ	Alcohol abuse = 7.8% Physical inactivity = 62.2%
						Unhealthy diet = 69.7%
						Dysglycemia = 7.1%
Enikuomehin et al. 2021 [104]	Nigeria (West Africa)	Healthcare	Descriptive cross-sectional	192	Hypertension = ≥140/90 mmHg	Hypertension = 22.4% Overweight = 26.0 %
						Obesity = 11.5%
					Dyslipidemia = NCEP panel guideline	Central obesity = 16.7%
					Unhealthy diet = No fruits and vegetables/day	Physical inactivity = 24%
					Physical inactivity = < 30 minutes/day	Unhealthy diet = 73.4%
						Dysglycemia = 3.1%
Etim et al. 2018 [105]	Nigeria (West Africa)	Healthcare	Descriptive cross-sectional	198	Stress assessment/workload analysis questionnaire	Stress = 92.8%
Owolabi et al. 2012 [106]	Nigeria (West Africa)	Healthcare	Descriptive cross-sectional	324	Hypertension = ≥140/90mmHg	Hypertension = 20.1%
					Job demand control questionnaire	Overweight = 24.7%
						Obesity = 9.9%
						Physical inactivity = 43.5%
						Alcohol intake = 6.5%
						Current tobacco smokers = 24.4%
						Stress = 26.2%
Capingana et al. 2013 [107]	Angola (Central Africa)	Education	Descriptive cross-sectional	615	Modified WHO-MONICA Project questionnaire	Hypertension = 45.2%
						Overweight = 29.3%
					Modified WHO STEPwise guidelines	Obesity = 19.6%
						Current tobacco smokers = 7.2%
					High TC = ≥ 240 mg/dl	Physical inactivity = 87.2%
						Diabetes = 5.7%
					High LDL-c = ≥ 160 mg/dl	High TC = 11.1%
						High LDL-c = 19.8%
					High TG = ≥ 150 mg/dl	Low HDL-c = 50.1%
						High TG = 10.6%
Mekonen et al. 2022 [108]	Ethiopia (East Africa)	Finance	Descriptive cross-sectional	285	Workplace stress assessment scale	Overweight = 20.0%
						Obesity = 5.3%
						Alcohol intake = 45.6%
						Current tobacco smokers = 1.1%
						Physical inactivity = 66.3%
						Stress = 21.1%
Aliyu et al. 2017 [109]	Nigeria (West Africa)	Healthcare	Descriptive cross-sectional	100	PSQI	Poor sleep = 61%
Agyei et al. 2019 [110]	Nigeria (West Africa)	Education	Descriptive cross-sectional	153	Effort-Reward Imbalance (ERI) Scale	Stress = 58.82%
Agyei et al. 2019 [110]	South Africa (Southern Africa)	Education	Descriptive cross-sectional	153	ERI Scale	Stress = 59.18%
Olatona et al. 2014 [111]	Nigeria (West Africa)	Finance	Descriptive cross-sectional	223	ISMA Questionnaire	Stress = 91.5%
Onyishi et al. 2022 [112]	Nigeria (West Africa)	Mixed	Descriptive cross-sectional	3,572	Depression, Anxiety, and Stress Scale- 21 items (DASS-21)	Stress = 94.13%
Chukwu 2023 [113]	Nigeria (West Africa)	Healthcare	Descriptive cross-sectional	270	Self-structured validated questionnaire	Stress = 86.3%
Makinde and Salawu 2021 [114]	Nigeria (West Africa)	Healthcare	Descriptive cross-sectional	196	Expanded Nursing Stress Scale (ENjSS)	Stress: 82.1%
Ogba 2020 [115]	Nigeria (West Africa)	Healthcare	Descriptive cross-sectional	337	Occupational stress index scale	Stress = 64.5%
Sime et al. 2022 [116]	Ethiopia (East Africa)	Manufacturing (Textile)	Descriptive cross-sectional	413	Workplace stress scale (WPSS)	Stress = 47.5%
						Alcohol intake = 37.0%
						Khat chewing = 12.1%
Bosu 2016 [117]	West Africa (West Africa)	Mixed	Systematic review	34,919	WHO STEPwise guidelines	Obesity:
						Healthcare = 42.1%
						Telecomm = 97.7%
Agbana et al. 2017 [118]	Nigeria (West Africa)	Agro-Allied	Nested case-control	510	WHO STEPwise guidelines	Stress = 44.1%
Awosan et al. 2013 [119]	Nigeria (West Africa)	Education	Quasi-experimental (Pre-test Post-test)	216	WHO STEPwise approach guidelines	Baseline:
						Hypertension = 29.6%
						Diabetes = 10.2%
						High TC = 89.8%
						Physical inactivity = 21.3%
						Current tobacco smokers = 3.7%
						Unhealthy diet = 68.5%
						Overweight = 28.7%
						Obesity = 13.0%
						Mean SBP = 115.84 mmHg
						Mean DBP = 78.02 mmHg
						Mean weight = 68.65 kg
						Mean FBS = 85.13 mg/dl
						Mean TC = 185.23 mg/dl
						Follow-up:
						Hypertension = 17.8%
						Diabetes = 3.0%
						High TC = 68.4%
						Physical inactivity = 6.9%
						Current tobacco smokers = 3.0%
						Unhealthy diet = 31.7%
						Overweight = 28.7%
						Obesity = 10.9%
						Mean SBP = 112.97 mmHg
						Mean DBP = 76.54 mmHg
						Mean weight = 67.64 kg
						Mean FBS = 78.44 mg/dl
						Mean TC = 172.52 mg/dl
Schouw et al. 2020 [120]	South Africa (Southern Africa)	Administration (Energy)	Randomized controlled trial	137	GPAQ	Baseline: alcohol intake = 78.2%
					AUDIT-10	Harmful alcohol intake = 21.8%
					Unhealthy diet = < 5 portions of fruits and vegetables/day	Current smoking = 25.0%
					PI = < 600 MET-minutes/week. 12-item stress screening tool	Unhealthy diet = 73.2%
						Physical inactivity = 55.9%
						Diabetes = 6.0%
						Hypertension = 15.9%
						High TC = 16.4%
						Stress level = 17.4%
						Mean SBP = 131.6 mmHg
						Mean DBP = 83.4 mmHg
						Mean TC = 5.6 mmol/l
						Mean RBS = 5.7 mmol/l
						Mean BMI = 29.0
						Mean waist circumference = 92.1 cm
						Follow-up:
						Alcohol intake = 93.5%
						Harmful alcohol intake = 6.4%
						Current tobacco smoking = 21.8%
						Unhealthy diet = 35.8%
						Physical inactivity = 34.7%
						Diabetes = 9.0%
						Hypertension = 18.9%
						High TC = 14.9%
						Stress level: 13.3%
						Mean SBP = 121.4 mmHg
						Mean DBP = 79.5 mmHg
						Mean TC = 5.1 mmol/l
						Mean RBS = 6.0 mmol/l
						Mean BMI = 29.0
						Mean waist circumference = 92.2 cm
Abiodun 2021 [121]	Nigeria (West Africa)	Administration	Randomized control trial	178	WHO STEPwise approach guidelines	Baseline:
						Unhealthy diet = 92.0%
						High salt intake = 45.4%
						Current smokers = 21.6%
						Passive smokers =27%
						Physical inactivity = 68.2%
						Mean BMI = 26.98
						Mean waist circumference = 90.0 cm
						Mean SBP = 125.43 mmHg
						Mean DBP = 80.48 mmHg
						Mean FBS = 5.32 mmol/l
						Mean TC = 4.97 mmol/l
						Total CVD Risk score = 5.45
						Follow-up:
						Unhealthy diet = 22.7%
						High salt intake = 5.7%
						Current tobacco smokers = 10.2%
						Passive smokers = 3.2%
						Physical inactivity = 29.5%
						Mean BMI = 25.20
						Mean waist circumference = 89.1 cm
						Mean SBP = 119.91 mmHg
						Mean DBP = 76.07 mmHg
						Mean FBS = 4.96 mmol/l
						Mean TC =4.56 mmol/l
						Total CVD Risk score = 5.11
Adelowo et al. 2020 [122]	Nigeria (West Africa)	Administration	Randomized control Trial	88	Finnish Diabetes Risk Score (FINDRISC) questionnaire	Baseline:
						Physical inactivity = 68.2%
						Unhealthy diet = 92%
						Mean BMI = 26.98
						Mean waist circumference = 90.0cm
						Diabetes Risk score = 7.82
						Follow-up:
						Physical inactivity = 29.5%
						Unhealthy diet = 22.7%
						Mean BMI = 25.2
						Mean waist circumference = 89.1 cm
						Diabetes Risk score = 6.06
Edries et al. 2013 [123]	South Africa (Southern Africa)	Manufacturing (Textile)	Randomized Control Trial	80	Health related Quality of Life (HRQoL EQ-5D) questionnaire	Increased Strength/stretch exercise = 70%
						Increased walking exercise = 77%
						Increased swimming exercise = 60%
					Stanford Exercise Behaviours Scale	Increased cycling exercise = 100%
						Increased in other aerobic exercises = 81%
						Reduction in BMI = 89%
Torres et al. 2020 [124]	South Africa (Southern Africa)	Finance	Randomized Control Trial	251	ACSM FITT-VP criteria	Baseline:
						Hypertension = 16.3%
						Obesity = 39.4%
						Physical inactivity = 55.4%
						Dyslipidemia = 6.4%
						Dysglycemia = 3.6%
						Current tobacco smokers = 3.6%
						Mean BMI = 28.2
						Mean waist circumference = 93.7 cm
						Mean SBP = 121.3 mmHg
						Mean DBP = 78.9 mmHg
						Follow-up:
						Hypertension = 10.8%
						Obesity = 32.7%
						Physical inactivity = 2.0%
						Dyslipidemia = 6.4%
						Dysglycemia = 3.6%
						Current tobacco smokers = 2.0%
						Mean BMI = 26.6
						Mean waist circumference = 91.5cm
						Mean SBP = 120.9 mmHg
						Mean DBP = 76.8 mmHg

Prevalence of Cardiovascular Risk Factors among the Corporate Workforce across Regions in Sub-Saharan Africa

**Figure 5:**
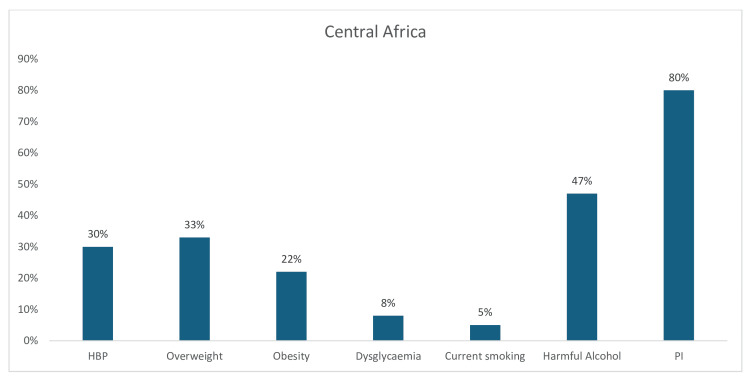
Graph showing the weighted prevalence of each risk factor across Central Africa The image was created by the authors of this article.

In Central Africa, PI (80%) was the leading risk factor, followed by harmful alcohol consumption (47%), overweight (33%), hypertension (30%), and obesity (22%). The prevalence of dysglycemia was only 8%, while the least recorded CV risk factor in Central Africa was current tobacco smoking, at only 5% (Figure 5).

**Figure 6:**
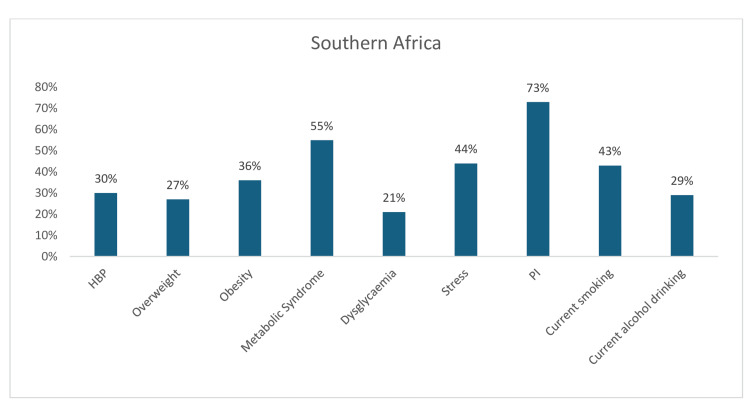
Graph showing the weighted prevalence of each risk factor across Southern Africa PI: physical inactivity The image was created by the authors of this article.

PI (73%) was also the leading risk factor in Southern Africa, followed by metabolic syndrome (55%), stress (44%), and current tobacco smoking (43%). Obesity (36%), hypertension (30%), current alcohol consumption (29%), overweight (27%), and dysglycemia (21%) (Figure 6).

**Figure 7:**
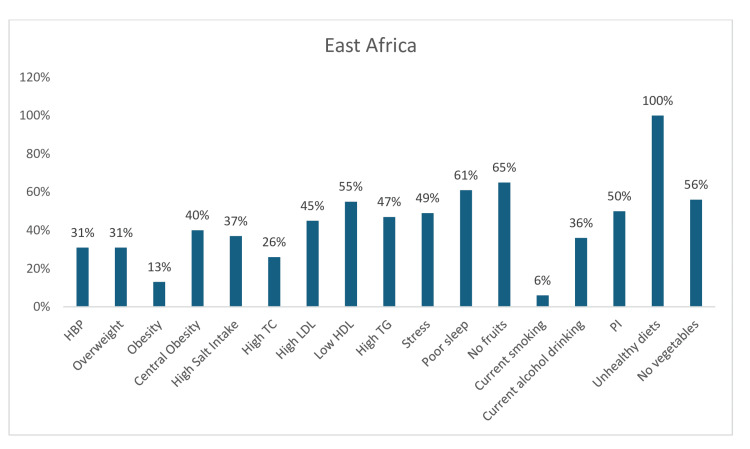
Graph showing the weighted prevalence of each risk factor across East Africa TC: total cholesterol; LDL: low-density lipoprotein; HDL: high-density lipoprotein; TG: triglyceride; PI: physical inactivity The image was created by the authors of this article.

Among the 17 CV risk factors reported across East Africa, the estimated prevalence of a generally unhealthy diet was universally high at 100%. Additionally, four other risk factors - poor sleep, no fruit consumption only, no vegetable consumption only, and low HDL-c-showed a notably high prevalence, ranging from 55% to 65%. The pooled prevalence of other components of dyslipidemia was also high (high triglycerides, 47%; high LDL-c, 45%; and high total cholesterol (TC), 26%). Furthermore, almost half (49%) of the participants had high-stress levels, 40% had central obesity, 44% were either overweight or obese, 37% regularly consumed high-salt diets, 36% currently drank alcohol, and 31% were hypertensive (Figure 7).

**Figure 8:**
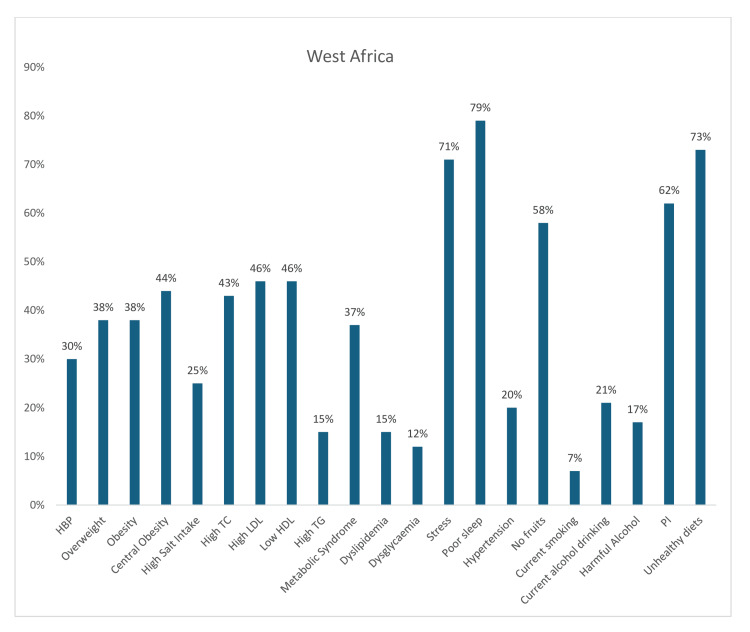
Graph showing the weighted prevalence of each risk factor across West Africa TC: total cholesterol; LDL: low-density lipoprotein; HDL: high-density lipoprotein; TG: triglyceride; PI: physical inactivity The image was created by the authors of this article.

In contrast, among the reported estimated prevalence of 21 risk factors among corporate employees in West Africa, there was a significantly high pooled prevalence of poor sleep (79%), stress (71%), general unhealthy diet (73%), and PI (62%). Also, 76% of the participants were either overweight or obese, while 44% had central obesity. In addition, there was a high prevalence of most components of dyslipidemia (high LDL-c, 46%; low HDL-c, 46%; high TC, 43%; and high triglyceride level, 15%) among West African workers. Furthermore, half (50%) of the participants had either a history of hypertension or hypertension on measurement, while more than one-third (37%) had metabolic syndrome (MS). On the other hand, only 21% drank alcohol, 17% drank alcohol to a harmful level, 12% had dysglycemia, and only 7% currently smoked tobacco products (Figure 8).

Prevalence of Cardiovascular Risk Factors among the Corporate Workforce across Countries in Sub-Saharan Africa

**Figure 9:**
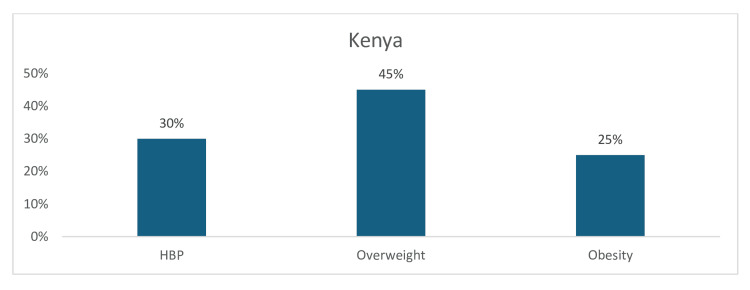
Graph showing the weighted prevalence of each risk factor in Kenya HBP: high blood pressure The image was created by the authors of this article.

The prevalence of the three cardiovascular risk factors was found to be common among studies conducted in Kenya. The results revealed a high pooled prevalence of 45% for overweight and a moderately high pooled prevalence of both obesity (25%) and hypertension (30%) (Figure 9).

**Figure 10:**
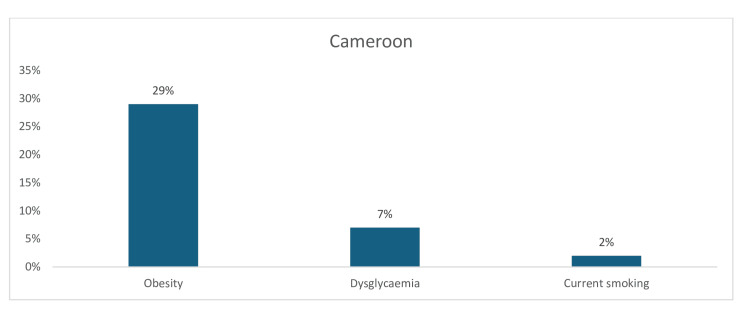
Graph showing the weighted prevalence of each risk factor in Cameroon The image was created by the authors of this article.

Three common CV risk factors have been identified in Cameroon. Here, the pooled prevalence of obesity (29%) was moderately high, followed at a far distance by dysglycemia (7%), whereas the pooled prevalence of current smoking was only 2% (Figure 10).

**Figure 11:**
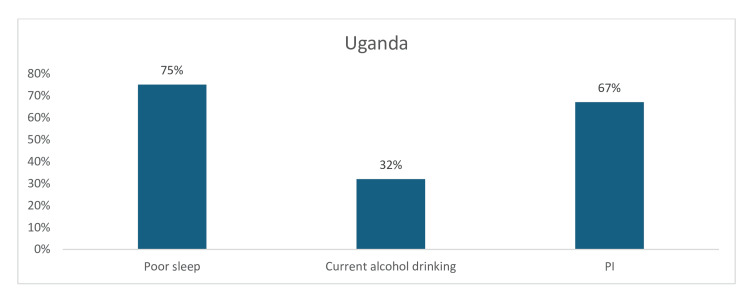
Graph showing the weighted prevalence of each risk factor in Uganda PI: physical inactivity The image was created by the authors of this article.

Similarly, among the CV risk factors reported in Uganda, poor sleep (75%) and PI (67%) were significantly higher, while current alcohol consumption was 32% (Figure 11).

**Figure 12:**
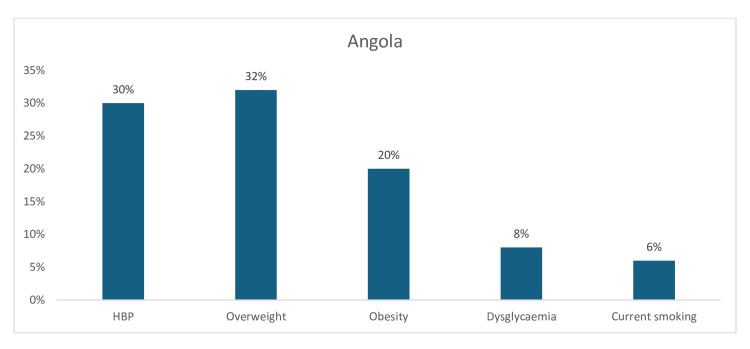
Graph showing the weighted prevalence of each risk factor in Angola HBP: High blood pressure The image was created by the authors of this article.

Moreover, in Angola, hypertension (30%) and overweight (32%) were reported to have a moderately high prevalence, followed closely by obesity (20%), while the pooled prevalence of dysglycemia (8%) and current tobacco smoking (6%) was low (Figure 12).

**Figure 13:**
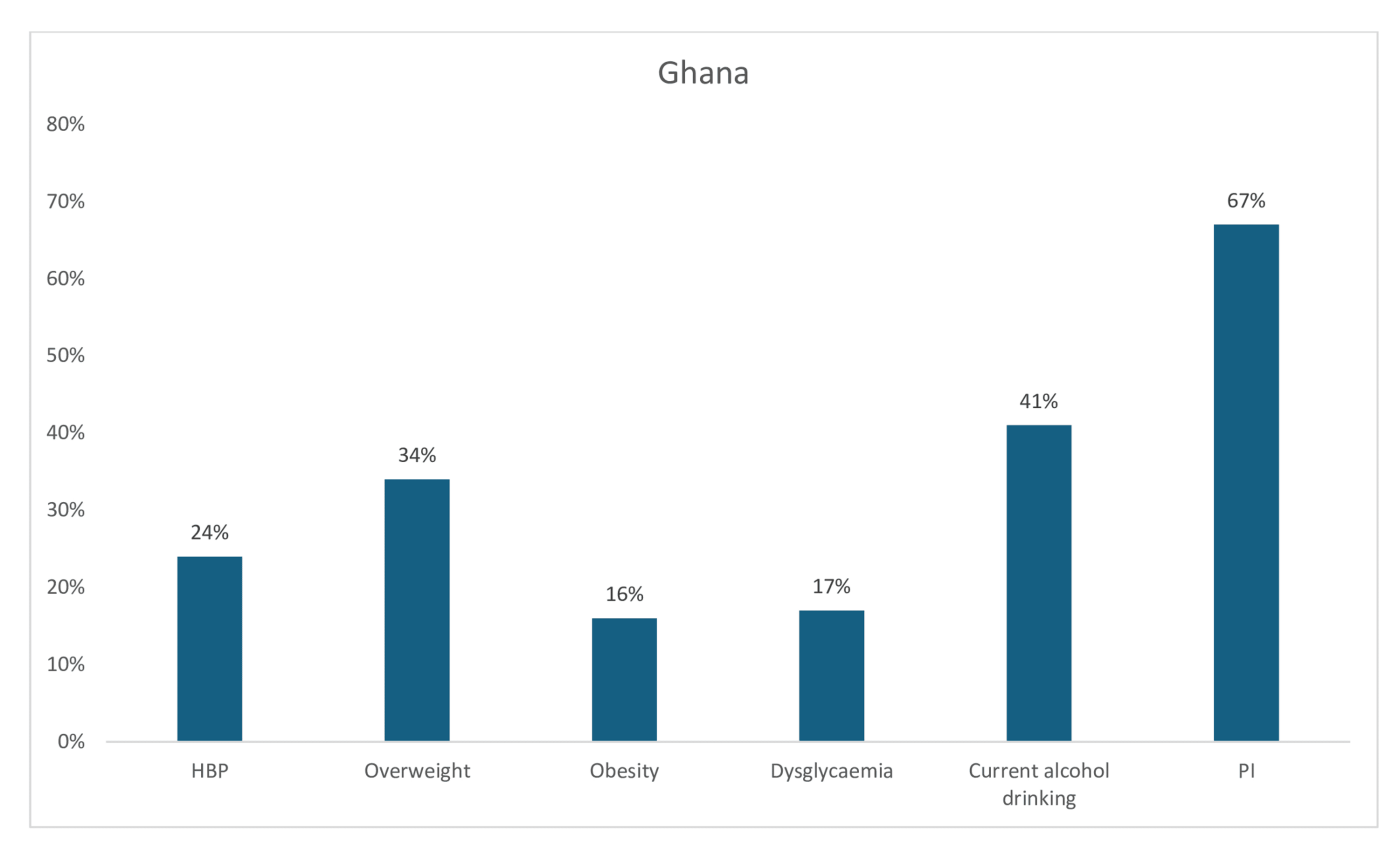
Graph showing the weighted prevalence of each risk factor in Ghana PI: physical inactivity; HBP: high blood pressure The image was created by the authors of this article.

In Ghana, PI (67%) and current alcohol consumption (41%) were highly prevalent among the reported risk factors, while the prevalence of overweight (34%), hypertension (24%), dysglycemia (17%), and obesity (16%) were moderately high (Figure 13).

**Figure 14:**
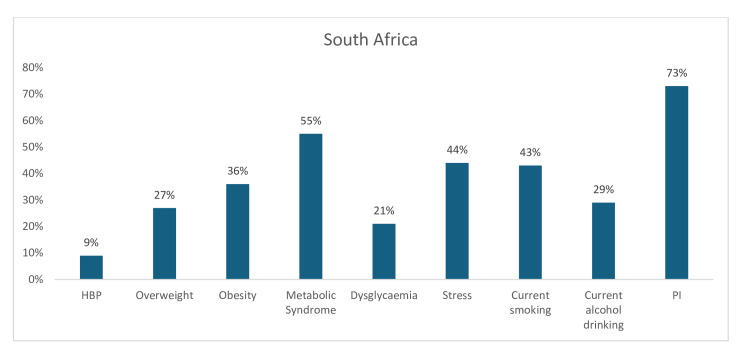
Graph showing the weighted prevalence of each risk factor in South Africa PI: physical inactivity; HBP: high blood pressure The image was created by the authors of this article.

Furthermore, this study identified nine common CV risk factors in South Africa. PI had the highest estimated prevalence of 73%, followed by MS at 55%. In addition, the prevalence of high BMI (overweight and obesity) was 63%, stress was 44%, current tobacco smoking was 43%, current alcohol intake was 29%, dysglycemia was 21%, and hypertension was 9% (Figure 14).

**Figure 15:**
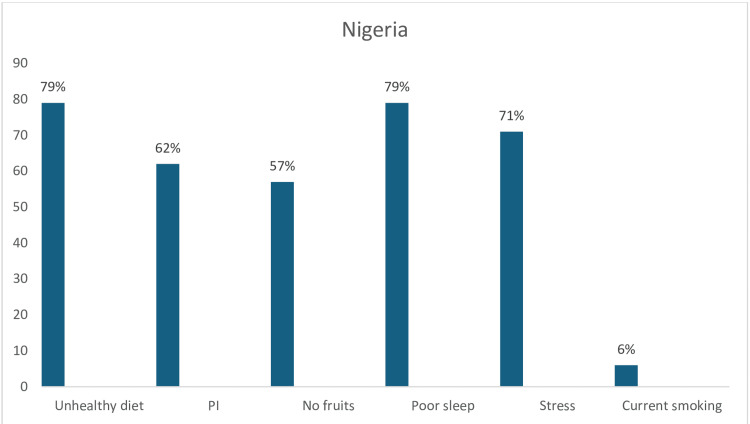
Graph showing the weighted prevalence of each risk factor in Nigeria PI: physical inactivity The image was created by the authors of this article.

In Nigeria, the observed CV risk factors were unhealthy diet (79%), poor sleep (79%), stress (71%), physical inactivity (62%), and no fruit consumption (57%), whereas only 6% currently smoked tobacco (Figure 15).

**Figure 16:**
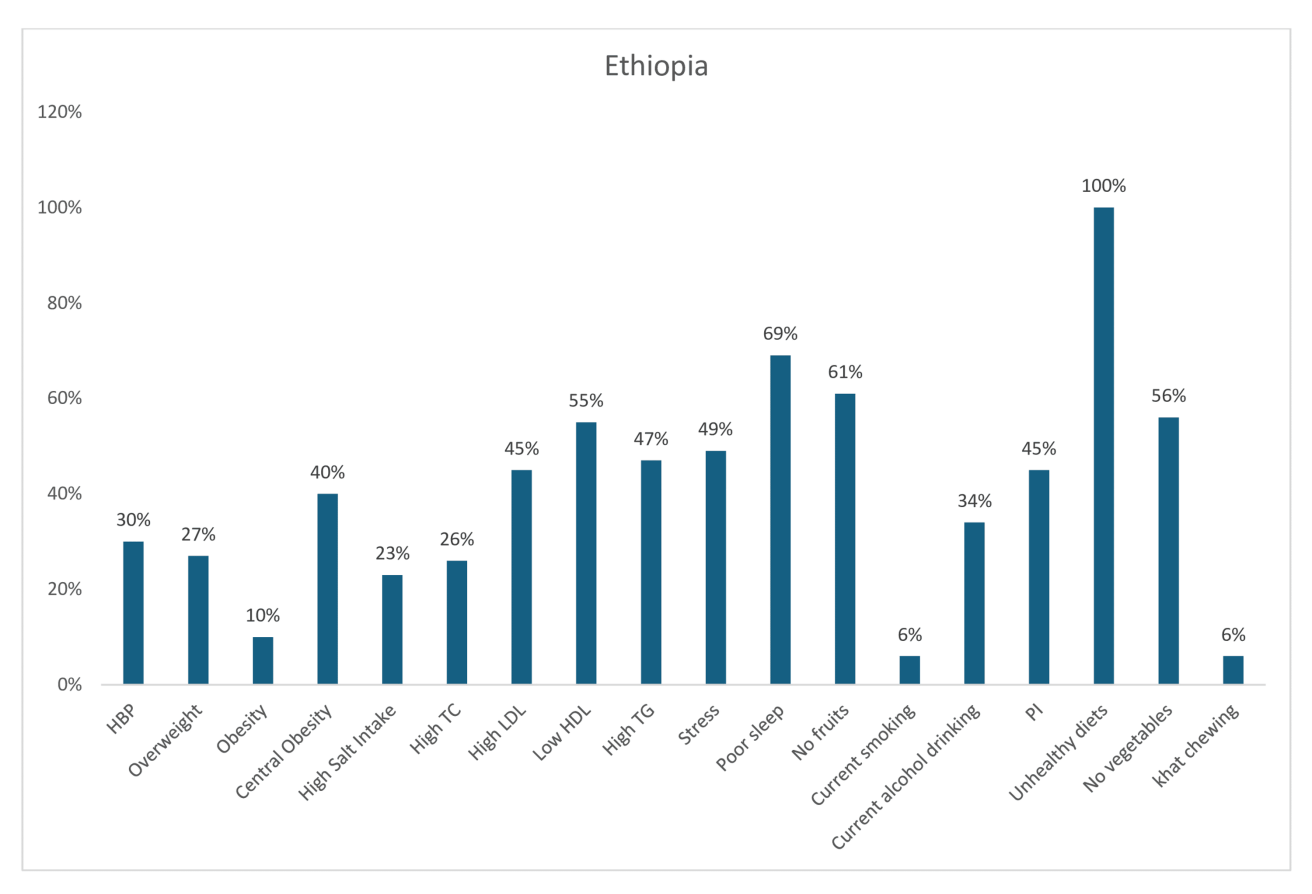
Graph showing the weighted prevalence of each risk factor in Ethiopia HBP: high blood pressure; TG: triglyceride; HDL: high-density lipoprotein; LDL: low-density lipoprotein; PI: physical inactivity The image was created by the authors of this article.

Similarly, in Ethiopia, a substantial number of participants regularly consumed a general unhealthy diet (100%), had poor sleep (69%), did not consume fruit only (61%), did not consume vegetables only (56%), and had low HDL levels (55%). The other CV risk factors that were noted in Ethiopia included stress (49%), hypertriglyceridemia (47%), high LDL-c (45%), physical inactivity (45%), central obesity (40%), current alcohol consumption (34%), hypertension (30%), overweight (27%), high TC (26%), high salt intake (23%), obesity (10%), current tobacco use/smoking (6%), and khat chewing (6%) (Figure 16).

Prevalence of Cardiovascular Risk Factors among the Corporate Workforce across Work Sectors in Sub-Saharan Africa

A systematic review was done on the eight distinct work sectors were noticed in the study, namely - administration, agro-allied, education, finance, healthcare, judiciary, manufacturing, and telecommunication. In addition, a systematic review was conducted across studies that reported the prevalence of CV risk factors in multiple work sectors (mixed).

**Figure 17:**
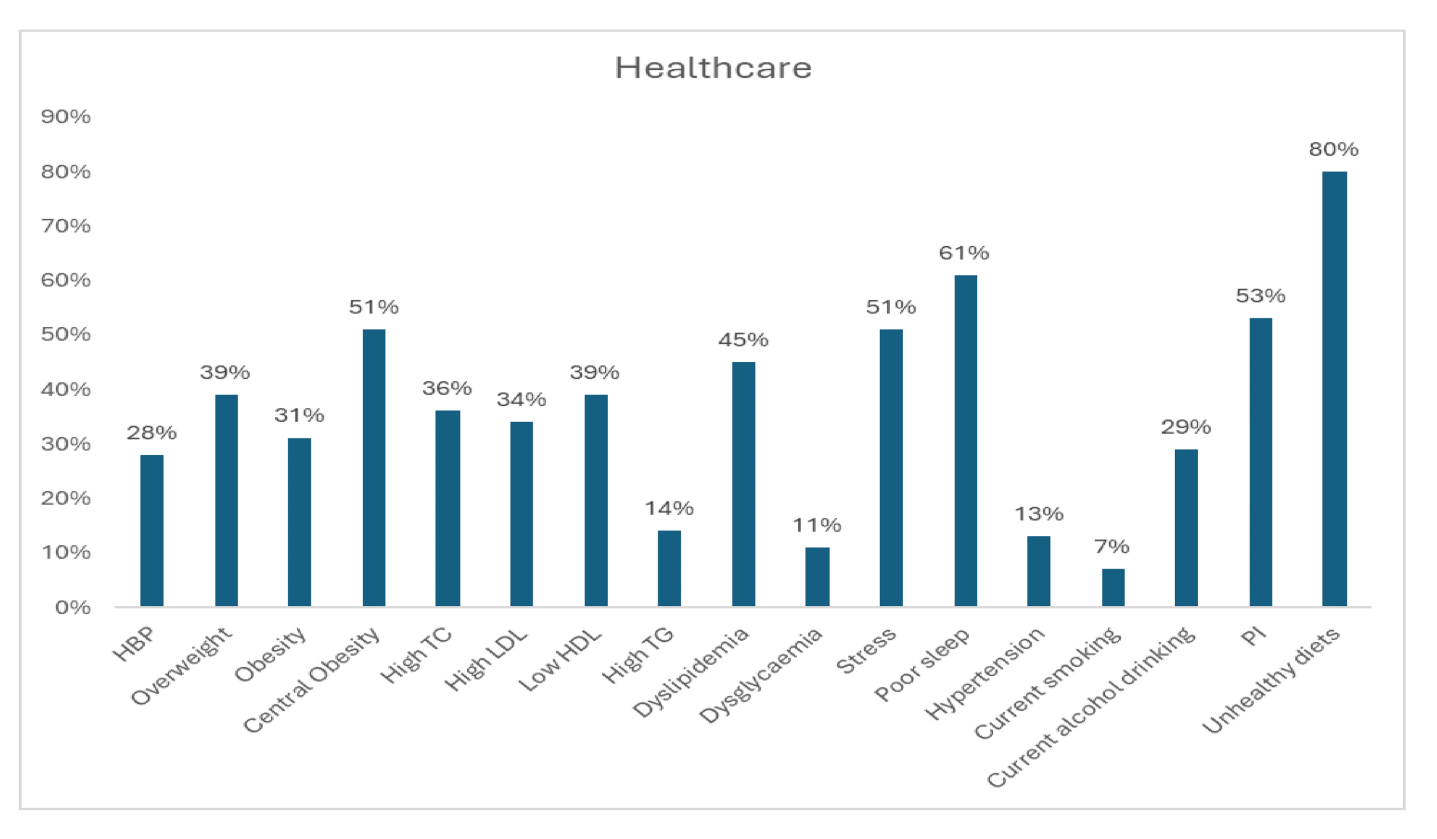
Graph showing the weighted prevalence of each risk factor in the healthcare sector HBP: high blood pressure; TG: triglyceride; HDL: high-density lipoprotein; LDL: low-density lipoprotein; PI: physical inactivity The image was created by the authors of this article.

Most risk factors were reported in the healthcare sector (17 risk factors), education (14 risk factors), and administration (10 risk factors). The most prevalent risk factors among healthcare workers were an unhealthy diet (80%), poor sleep (61%), PI (53%), central obesity (51%), and stress (51%). The prevalence of central obesity, dyslipidemia, and hypertension (previous history of hypertension and hypertension on measurement) was 51%, 51%, and 41%, respectively. Various components of dyslipidemia (low HDL-C, 39%; high TC, 36%; high LDL-C, 34%; and hypertriglyceridemia, 14%) were also moderately high. The other risk factors that were noticed among healthcare workers included dyslipidemia (45%), overweight (39%), obesity (31%), current alcohol drinking (29%), dysglycemia (11%), and current tobacco use/smoking (7%) (Figure 17).

**Figure 18:**
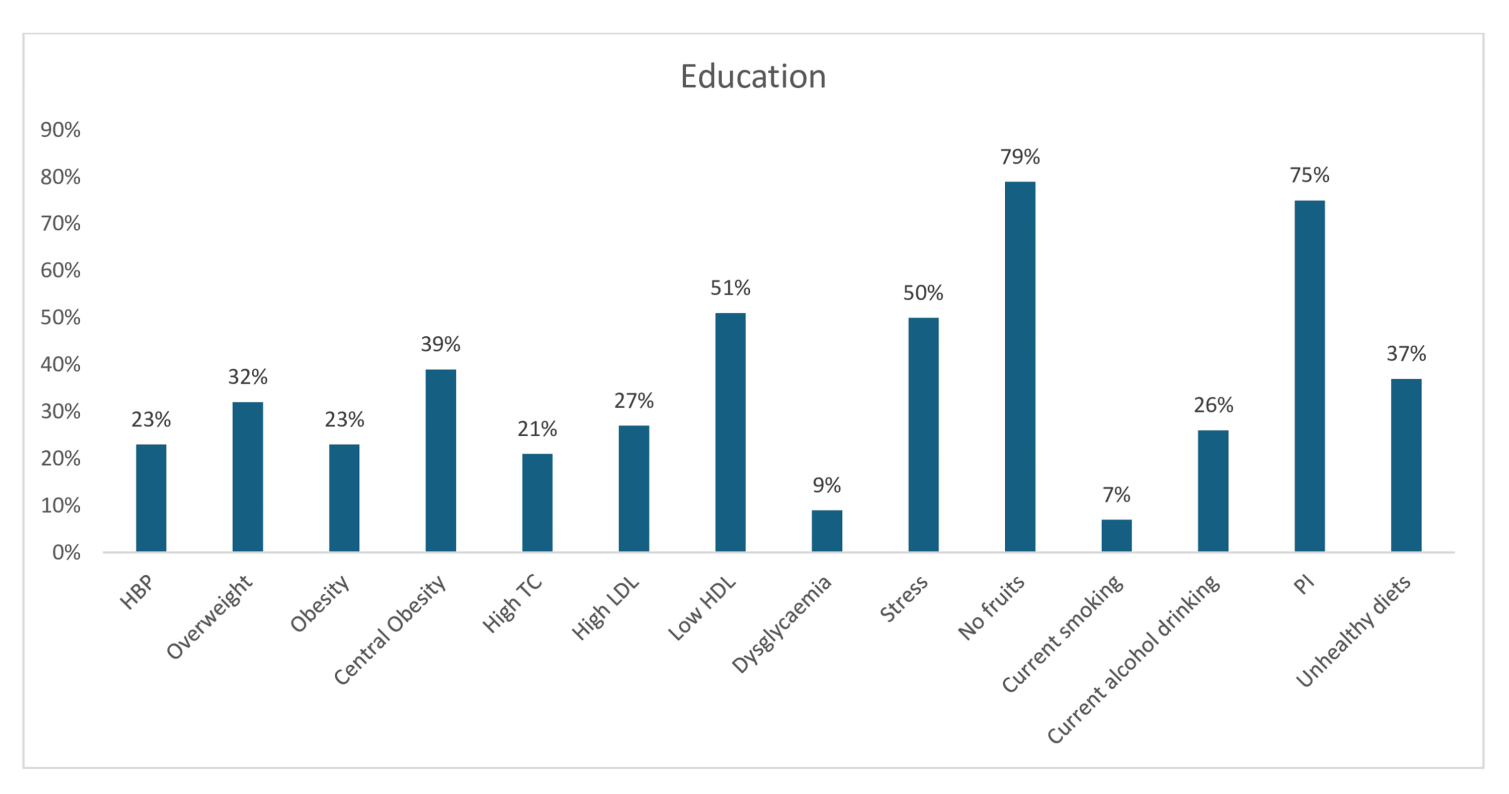
Graph showing the weighted prevalence of each risk factor in education sector HBP: high blood pressure; TG: triglyceride; HDL: high-density lipoprotein; LDL: low-density lipoprotein; PI: physical inactivity The image was created by the authors of this article.

On the other hand, among academia/educators, the most prevalent risk factors were physical inactivity (75%), no fruit consumption only (79%), stress (50%), and low HDL (51%). The other CV risk factors that were noticed in the academia/education sector included central obesity (39%), unhealthy diet (37%), overweight (32%), current alcohol intake (26%), obesity (23%), hypertension (23%), high LDL-c (27%), high TC (21%), dysglycemia (9%), and current tobacco use/smoking (7%) (Figure 18).

**Figure 19:**
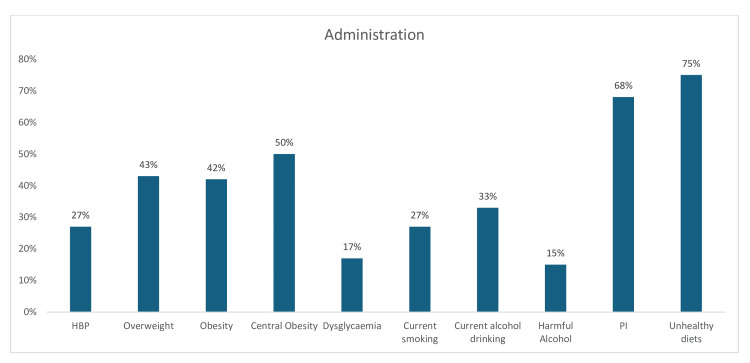
Graph showing the weighted prevalence of each risk factor in Administration sector PI: physical inactivity; HBP: high blood pressure The image was created by the authors of this article.

In the administration sector, the prevalence of unhealthy diet (75%), PI (68%), and central obesity (50%) were estimated to be higher than those of other risk factors in the sector. This was followed by being overweight (43%) and obese (42%). The prevalence of other CV risk factors was as follows: current alcohol intake (33%), hypertension (27%), current tobacco use/smoking (27%), dysglycemia (17%), and harmful alcohol intake (15%) (Figure 19).

**Figure 20:**
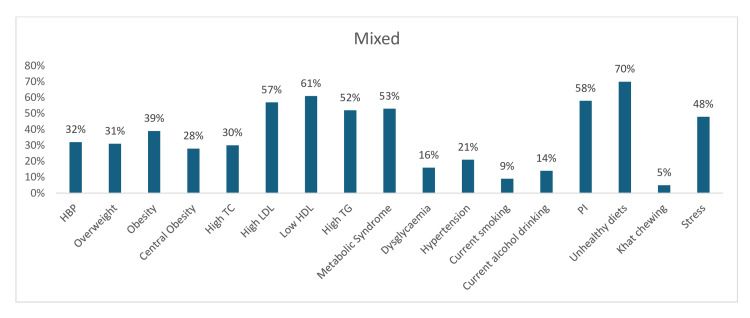
Graph showing the weighted prevalence of each risk factor in the mixed sectors HBP: high blood pressure; TG: triglyceride; HDL: high-density lipoprotein; LDL: low-density lipoprotein; PI: physical inactivity; TC: total cholesterol The image was created by the authors of this article.

Furthermore, the study showed a high estimated prevalence of an unhealthy diet (70%), physical inactivity (58%), metabolic syndrome (53%), low HDL (61%), high LDL (57%), and high TG (52%) among the mixed sectors (Figure 20).

**Figure 21:**
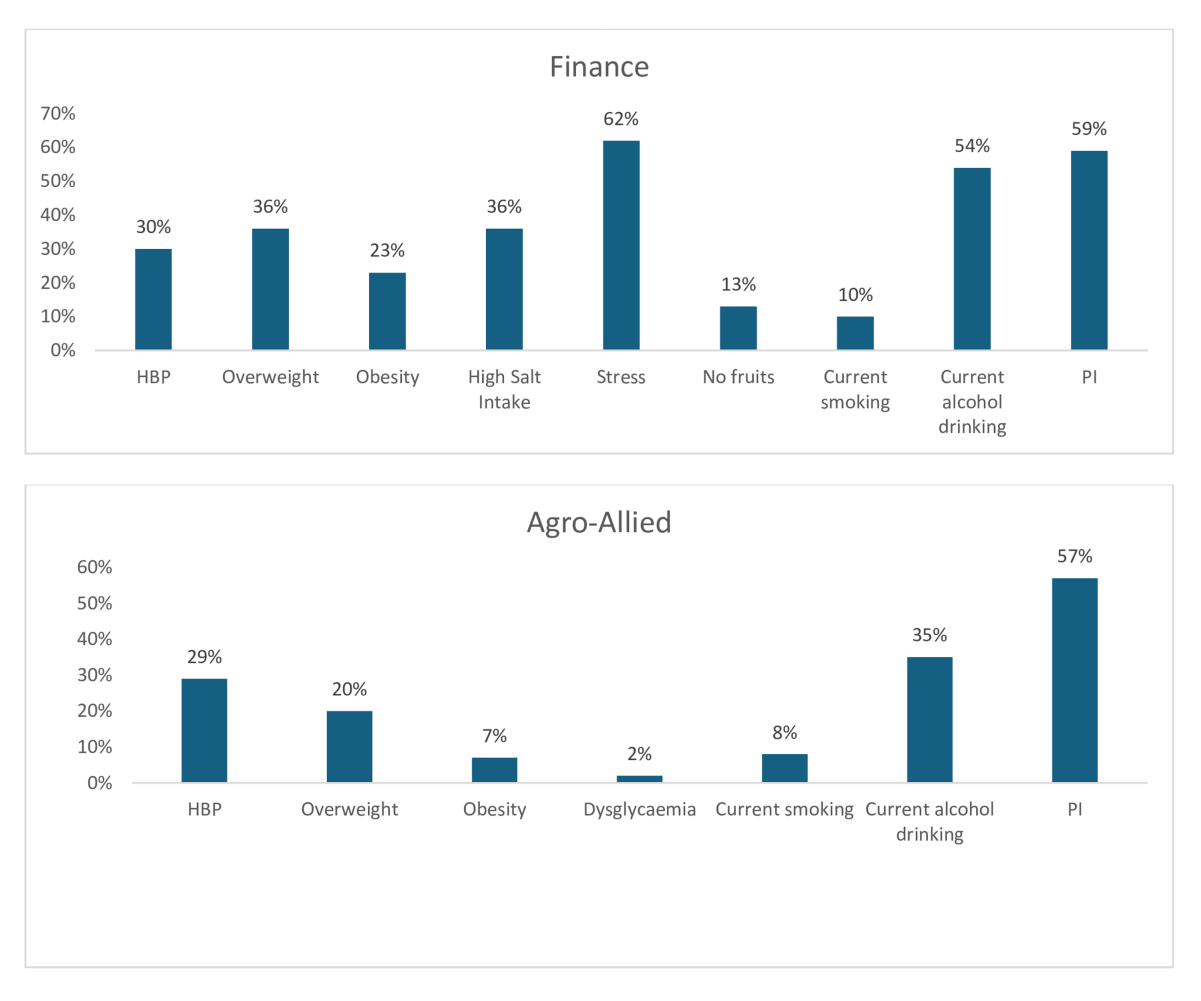
Graphs showing the weighted prevalence of each risk factor in Finance sector and the weighted prevalence of each risk factor in agro-allied sector PI: physical inactivity; HBP: high blood pressure The image was created by the authors of this article.

Conversely, stress (62%) was the most prevalent risk factor among the financial sector workers, this is closely followed by PI (59%), then current alcohol consumption (54%). Furthermore, high salt intake (36%), overweight (36%), hypertension (30%), and obesity (23%) were moderately high among financial sector workers, while the prevalence of no fruit intake only and current tobacco smoking was low at 13% and 10%, respectively (Figure 21). In the agro-allied sector, PI (57%) was the most prevalent CV risk factor, followed by current alcohol consumption (35%), hypertension (29%), and being overweight (20%). However, the prevalence of obesity (7%), current tobacco use/smoking (7%), and dysglycemia (2%) was low among agro-allied sector workers (Figure 21).

**Figure 22:**
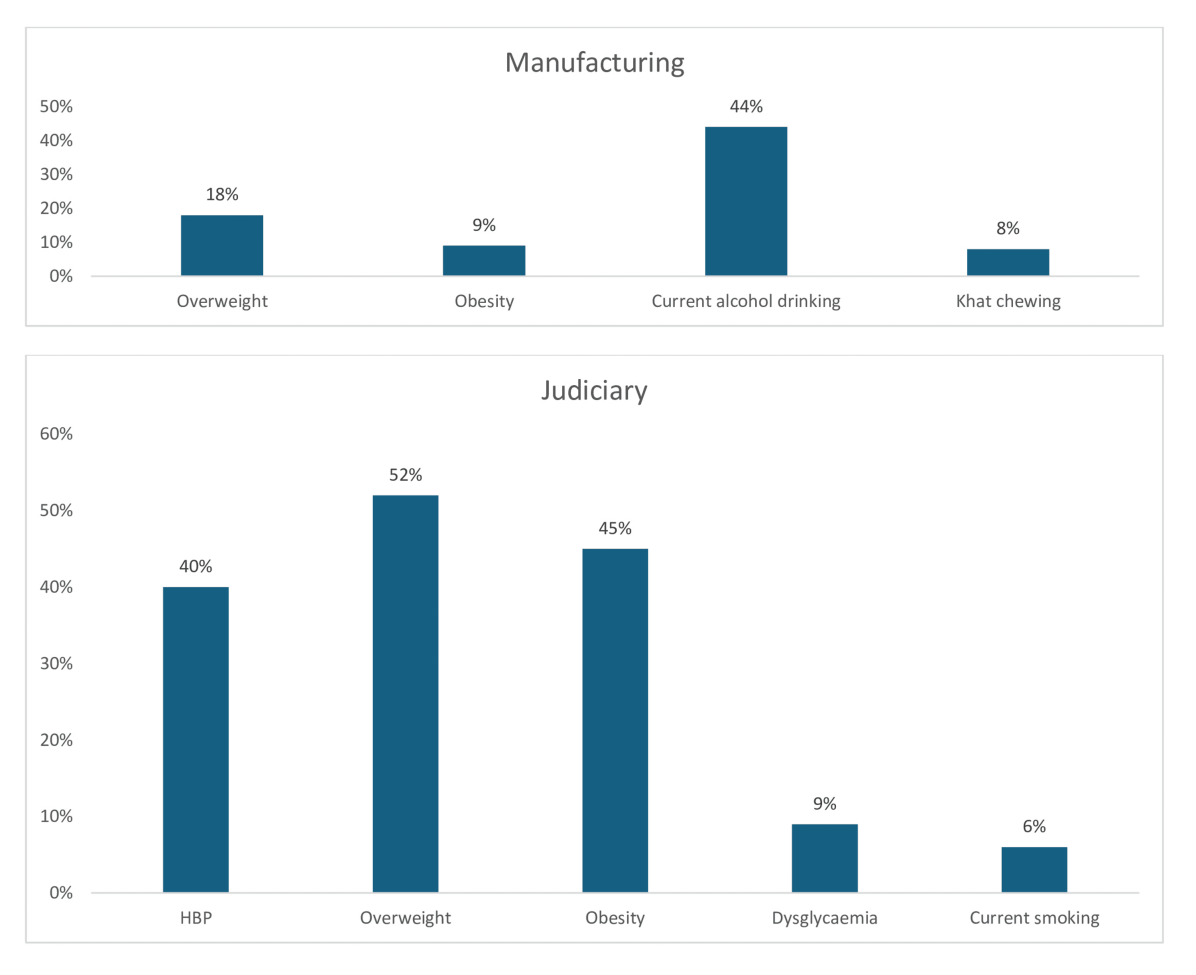
Graph showing the weighted prevalence of each risk factor in manufacturing sector and in the judiciary sector HBP: high blood pressure The image was created by the authors of this article.

**Figure 23:**
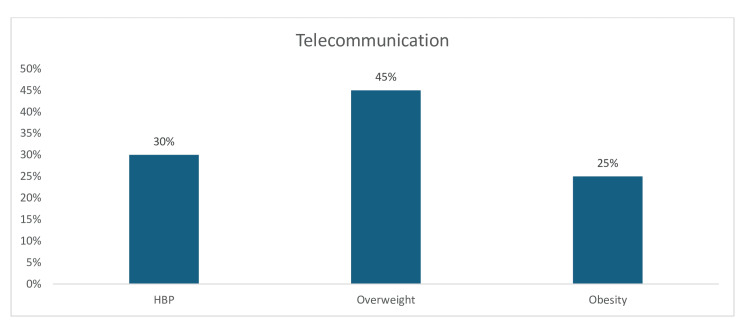
Graph showing the weighted prevalence of each risk factor in Telecommunication sector HBP: high blood pressure The image was created by the authors of this article.

Current alcohol consumption (44%) was the most prevalent CV risk factor among manufacturing sector workers, followed by overweight (18%) and obesity (9%), while 8% of manufacturing workers also chew khat (Figure 22). Furthermore, in the judiciary sector, the three most prevalent CV risk factors are overweight (52%), obesity (45%), and hypertension (40%). However, the prevalence of dysglycemia (9%) and current smoking (6%) was low among judiciary workers (Figure 22). Finally, in the telecommunications sector, the prevalence of overweight (45%) was high, whereas the prevalence of hypertension (30%) and obesity (25%) was moderately high (Figure 23).

Discussion

Prevalence of Behavioral Risk Factors among the Corporate Workforce across the Regions in Sub-Saharan Africa

Multiple studies have associated some behavioral or lifestyle-related factors with increased risk or severity of CVD. These include unhealthy diet, PI, stress, poor sleep, alcohol consumption/abuse, tobacco use, and khat chewing [124-128]. An unhealthy diet can increase the risk and burden of CVD through multiple pathomechanisms, such as by causing significant systemic chronic inflammation (SCI) and increasing plague formations in the blood vessels [129]. PI can increase the risk of coronary heart disease (CHD) or stroke by 30% to 50% [15], directly by reducing the efficacy of the heart muscle and causing plague formations in the blood vessels [130,131], and indirectly, increasing the risk of developing some other CVD risk factors, such as obesity, hypertension, dyslipidemia, and diabetes [132].

On the other hand, chronic stress can increase the risk or severity of CVD directly by increasing some cardiovascular system damaging factors, such as SCI, C-reactive protein level, and activity in the amygdala of the brain [133], and indirectly, by increasing the prevalence of other CVD risk factors, such as hypertension, diabetes, PI, unhealthy diet, increased substance abuse, and social isolation/loneliness [133,134]. Also, poor sleep can directly result in an increased SCI, cortisol levels, atherosclerosis formation, and disruption in the integrity and functionality of the cardiometabolic systems [135-137]. Poor sleep can also increase the prevalence of obesity, unhealthy diet, hypertension, stress, dyslipidemia, dysglycemia, and substance abuse [15,135,137].

Alcohol consumption can increase the prevalence of some cardiovascular system damaging factors, such as hypertension, obesity, and high cortisol level [138-140]. Furthermore, tobacco use can directly increase the atherosclerotic process in the blood vessels [141-143], and indirectly increase the risk of CVD by increasing coagulation, blood pressure, dyslipidemia, and tachycardia [141,143,144].

The majority (69%) of the included studies were conducted in West Africa, followed by East Africa (14%), and Sothern Africa (12%), while Central Africa contributed only 4% of the study. In the same pattern, Nigeria (a West African country) contributed the most studies (68) to this systematic review. The reason for this disproportionate outcome is not obvious. It may however be because Nigeria is the most populous black nation in Africa and the world [145], with a literacy rate as high as 77.62% in 2021 [146]. This may translate to many people in the country having a higher appetite for research.

With regard to behavioral risk factors, Central Africa (80%) had the highest pooled prevalence of PI, followed by Southern Africa (73%), West Africa (62%), and East Africa (50 %). It is instructive to note that at least half of the corporate workforce in all SSA regions does not meet the minimal WHO requirements for adequate physical activity.

It is not apparent why the prevalence of PI among the corporate workforce in SSA was high or why the distribution of PI across the different regions in SSA followed this pattern. However, the results are similar to the 2021 WHO Global Health Observatory report which documented the prevalence of insufficient physical activity among adults in 42 African countries. According to the WHO report, Cameroon (one of the two Central African countries that contributed articles for this study) was ranked 8th, with the highest prevalence of insufficient PI in Africa, while Uganda (one of the two East African countries that contributed articles for this study) was ranked the most physically active country in Africa.

Furthermore, two other East African countries (Kenya and Ethiopia) that contributed articles for this study were ranked 30th and 32nd, respectively, and the lowest countries in Africa with insufficient physical activity [125]. Thus, the results of this study, in collaboration with the WHO, suggest that East Africans are generally more active, while Central Africans are generally less active than other African regions. Regardless, the high prevalence of PI among the corporate workforce across all regions in SSA is concerning. This is because the results of a recent survey across Africa suggest that 79.1% and 72.8% of the general African population met the criteria of the WHO and the GPAQ (Global Physical Activity Questionnaire) respectively, for sufficient physical activity [126]. Thus, it seems working in a corporate workplace is significantly reducing the physical activity level of sub-Saharan Africans in all the regions.

For alcohol consumption, the result of this study suggests that the corporate workforce in East Africa (36%) drinks alcohol the most, this is followed by Southern Africa (29%), and the least is West Africa with 21%. However, the corporate workforce in Central Africa (47%) engaged in harmful alcohol consumption the most, followed by West Africa (17%). This pattern is somewhat congruent with the suggestions of the 2024 WHO Global Status Report on alcohol, health, and treatment of substance use disorders. According to a WHO report, Uganda (an East African country) was ranked the country in Africa with the highest alcohol per capita (APC) consumption, while South Africa (the only Southern African country that contributed articles to this study) was ranked 6th highest alcohol per capital-consuming nation in Africa [127].

The WHO report further informed that Angola and Cameroon (two Central African countries that contributed articles to this study) ranked 2nd and 8th highest on the list of alcohol-heavy episodic drinking nations in Africa [127]. Thus, as suggested by this study and in collaboration with the WHO, it seems that people in East and Southern African countries consume more alcohol than the other SSA regions, while Central Africans engage in heavy alcohol consumption more than the other SSA regions. Therefore, the pattern of alcohol consumption noticed among the corporate workers in this study may be due to cultural influences from different regions.

More than three-quarters (79%) of the corporate workforce in West Africa are experiencing poor sleep, and another 71% are stressed. The region is followed by East Africa with 61% poor sleep and 49% stress, while Southern Africa had 44% stress level. This result corroborated the position of the 2024 Gallup’s report, which stated that about half (46%) of SSA workers experienced stress every day [128]. There is an inverse and bidirectional relationship between stress and sleep [129]. This might explain the reason why the region with the highest prevalence of poor sleep also recorded the highest prevalence of stress. It is not completely apparent why the corporate workers in West Africa seem to be more stressed and getting less sleep compared to other regions. However, this might be related to the current high economic inflation rate in many countries in West Africa. Based on the 2024 report of Trading Economics, Nigeria and Ghana (the two West African countries that contributed articles to this study) are currently ranked 3rd and 9th among the nations with the highest economic inflation rate in Africa [130]. Since high inflation may result in low purchasing power, slower economic growth and job expansion, and high worker layoffs [131,132], this may be one of the reasons, in addition to unhealthy work culture and environment, which causes a high prevalence of stress and poor sleep among corporate workers in West Africa. Other factors that may contribute to the high prevalence of stress and poor sleep among the workforce in West Africa might be the high rates of insecurity and poor infrastructural development present in some countries in West Africa. The high prevalence of stress and poor sleep across the corporate workforce in SSA is worrisome and should be comprehensively addressed. It is instructive to note that almost half (43%) of the participants in Southern Africa currently uses tobacco products, West Africa (7%) came a far distant second, followed by East Africa (6%), and the least is Central Africa (5%). The observed relatively low prevalence of tobacco use/smoking in all the regions in SSA, except Southern Africa, is supported by many studies that reported that Africa presently experiences the lowest prevalence of tobacco smoking in the world [133]. However, the prevalence of tobacco use is increasing progressively in SSA, and it seems that the Southern African region has led to a negative change. The findings of this study are somewhat congruent with the estimation of the 2024 WHO global report on tobacco use, which ranked many Southern African countries on the prevalence list of current tobacco smoking nations in Africa [134].

According to the 2024 WHO report, South Africa (the only Southern Africa that contributed articles to this study) had a current tobacco smoking prevalence of 20.3%, which makes her the 5th country with the highest current tobacco smoking nation in Africa [133]. Other Southern African countries, such as Lesotho (22.9%) and Botswana (18.1%) were ranked 3rd and 7th countries respectively, with the highest prevalence of current tobacco smoking in Africa [133]. On the other hand, Cameroon (one of the two Central African countries that contributed articles to this study) was ranked by the WHO as the 5th country with the lowest prevalence of current tobacco smoking in Africa [133]. In addition, the results of a recent study also indicated that West Central African men have the lowest cigarette smoking rate in SSA [135]. Thus, as suggested by this study and supported by the WHO and the results of other studies, it seems that the prevalence of current tobacco smoking is generally high among many Southern African countries and low among many Central African countries compared to other SSA regions. Therefore, the regional distribution of current tobacco smoking that was observed among the participants in this study may be due to cultural influences from different regions. Some of the possible reasons for the high prevalence of tobacco use among people in the Southern African regions include favorable prices, a high youth population (especially males), a marketing strategy that encourages single-stick sales, culture and social norms that encourage cigarette sharing, and high levels of addiction to tobacco products among people in this region [136].

It is worth noting that 100% and almost three-quarters (73%) of the corporate workforce in East Africa and West Africa, respectively, do not meet the minimum recommendations for a healthy diet, while 65% and 56% of the participants in East Africa consume no fruit per day and or vegetables per day, respectively. Also, 37% and 25% of the participants in East Africa and West Africa consume salt in excess. This finding is supported by the results of multiple studies and reports which indicate that the consumption of vegetables is generally low among most East African countries [137-139]. Studies have also shown that the prevalence of zero vegetable and fruit (ZVF) intake can be as high as 56.1% in both West and Central African regions [138]. Thus, cultural influences may have played a significant role in the unhealthy diet pattern observed in this study. Some of the possible reasons that have been suggested for the poor consumption of vegetables and fruits in East Africa and some regions in SSA include low purchasing power, scarcity of some vegetables and fruits, socioeconomic variation, environmental changes, low household and community wealth indices, and low literacy levels [139]. It is also worth noting that khat chewing was noticed only in the East African corporate workforce. This finding also suggests a strong cultural practice of khat chewing in East Africa.

Prevalence of Intermediate Risk Factors among the Corporate Workforce across the Regions in Sub-Saharan Africa

The corporate workforce in West Africa had the highest prevalence of high BMI at 76% (38% for overweight and 38% for obesity), they also had the highest prevalence of central obesity (44%). This is closely followed by the Southern Africa region with a high BMI of 63% (27% for overweight and 36% for obesity), then by Central Africa with a high BMI of 55% (33% for overweight and 22% for obesity). The least is East Africa with a high BMI of 44% (31% for overweight and 13% for obesity). However, it is instructive to note that although East Africans had the lowest prevalence of high BMI, their pooled prevalence of central obesity was significantly high at 40%. The results of this study contrast the results of other studies that found that Southern Africans are more obese than the rest of the regions [140,141].

This result is worrisome because, except for East Africa, at least 50% of the workers in other regions of SSA are either overweight or obese. According to the WHO’s Global Health Observatory, SSA houses many countries with the lowest BMI in the world [142]. This means that the prevalence of overweight and obesity is higher among the corporate workforce in SSA than among the general population in the region. Thus, this result buttresses the position that an unhealthy workplace and environment in many organizations may increase the overweight/obesity prevalence among workers [16,143].

Another possible reason for the high prevalence of overweight and obesity in West and Southern Africa is the rising phenomenon of urbanization and westernization in these regions and many communities in Africa [144]. These phenomena encourage sedentary lifestyles (such as frequent vehicular transportation) and frequent consumption of energy-dense foods [144]. A situation that may have been worsened by unhealthy workplace culture and environment encourages prolonged sitting (with insufficient time to exercise) and other unhealthy lifestyle practices [145-147].

According to this study, all the regions had a pooled prevalence of 30% for hypertension, except East Africa with a slightly higher prevalence of 31%. It is worth noting that most of the included studies only measured the blood pressure of their participants to determine who was hypertensive. Most did not ask about the history of hypertension or the use of antihypertensives in their participants. This might explain why all regions had a moderately high prevalence of hypertension. Most of the studies that took the history of hypertension in their participants in this study are from West Africa, where the prevalence of previous history of hypertension among the study participants was 20%. This suggests that the prevalence of hypertension in the corporate workforce in West Africa is approximately 50%. Thus, the prevalence of hypertension may have been underestimated in other regions.

The findings of this study are different from those of a recent Africa-wide study that noticed that the age-adjusted pooled prevalence of hypertension was highest in West Africa, followed by South Africa, North Africa, and East Africa [141]. In addition, another study estimated that the prevalence of hypertension in Nigeria, Guinea Bissau (West African countries), and some Southern Africa (such as South Africa and Namibia) is more than 35%, which is higher than the prevalence of hypertension in most other countries in Africa [148]. Similar to obesity, increasing urbanization and westernization have been implicated as reasons for the increasing prevalence of hypertension in West and Southern Africa and other regions of Africa [148].

The prevalence of dysglycemia was highest in Southern Africa (21%), followed by West Africa (12%), and then by Central Africa (8%). Also, the Southern Africa region (55%) had a higher prevalence of MS compared to the West African region (37%). The high prevalence of some behavioral risk factors, such as PI, alcohol consumption, and tobacco smoking, may be one of the reasons why the prevalence of dyslipidemia and metabolic syndrome is higher in Southern Africa than in other regions.

Only the studies from West Africa and East Africa investigated the prevalence of dyslipidemia among their corporate workforces in this study. Out of these, the prevalence of high TC and high LDL-c was higher in West Africa (high TC was 43% and high LDL-c was 46%) compared to East Africa (high TC was 26% and high LDL-c was 45%). On the other hand, the prevalence of low HDL-c and hypertriglyceridemia (TG) was higher in East Africa (low HDL-c was 55% and high TG was 47%) compared to West Africa (low HDL-c was 46% and high TG was 15%). The high prevalence of some behavioral and intermediate risk factors, such as unhealthy diet, alcohol consumption, poor sleep, stress, obesity, and central obesity may explain the reason why there is a high prevalence of dyslipidemia in both West and East Africa.

Prevalence of Cardiovascular Risk Factors among the Corporate Workforce across Countries in Sub-Saharan Africa

The studies included in this systematic review were selected from nine countries across the four sub-regions of SSA. In Kenya, the prevalence of overweight and obesity is 45% and 25%, respectively, while the prevalence of hypertension is 30% among corporate workers. These findings are far higher than the results of a recent national survey in Kenya, where the prevalence of overweight and obesity was 21% and 10%, respectively, whereas the prevalence of hypertension was only 6% [149].

In addition, according to the 2021 joint scorecard of the Pan-African Society of Cardiology (PASCAR) and the World Heart Federation (WHF), as of 2015, the prevalence of overweight and obesity among Kenyan adults (18-69 years) was only 19% and 8.9%, respectively [150]. Thus, the findings of this study suggest that the prevalence of high BMI and hypertension was higher among the corporate workforce in Kenya compared to the general Kenyan population.

In Cameroon, the pooled prevalence of obesity was 29% among corporate workers. This result is higher than the WHO country estimated 10% obesity prevalence in Cameroon [150]. However, the pooled prevalence of current smoking in Cameroon in this study was only 2%, which is in agreement with the WHO country estimated 4.6% current smoking prevalence [134]. Thus, the finding of this study suggests that the corporate workforce in Cameron is more obese than the general population in the country. In addition, the low prevalence of tobacco use/smoking in Camerron may be due to cultural influences from the general population.

In Uganda, there was a significantly high prevalence of poor sleep (75%) and PI (67%), while the pooled prevalence of current alcohol drinking was moderately high at 32% among corporate workers. This result is far higher than the WHO’s country estimate of just 5% prevalence of PI in Uganda [151]. However, this result is lower than the WHO country-estimated 42.6% prevalence of current alcohol drinkers in Uganda [127]. Thus, the finding of this study suggests the Ugandan workforce is grossly more physically inactivity compared to the general population.

The pooled prevalence of overweight, obesity, hypertension, and current smoking among Angolan corporate workers was 32%, 20%, 30%, and 6%, respectively. These results are far higher than the WHO’s estimated prevalence of 7% and 22% for obesity and hypertension, respectively in Angola [151]. Thus, the findings of this study suggest that the corporate workforce in Angola has a higher prevalence of obesity and hypertension than the general population in the country.

In this study, the prevalence of PI and current alcohol consumption among Ghanian corporate workers was significantly high, at 67% and 41%, respectively. In addition, the prevalence of obesity and hypertension was moderately high, at 16% and 24%, respectively. It is also instructive to note that 17% of the corporate workforce in Ghana is already either prediabetic or diabetic. These results are far higher than the WHO’s country estimates of prevalence of 20%, 19%, 10%, and 5% for PI, hypertension, obesity, and diabetes, respectively, for Ghana [151]. The result is, however, in collaboration with the WHO’s estimate of 42.8% prevalence of current alcohol consumption in Ghana [127]. Thus, the findings of this study suggest that the prevalence of many CV risk factors is significantly higher among the Ghanaian corporate workforce than among the general population in the country and that the prevalence of alcohol consumption among the Ghanian workforce may be culturally influenced.

This study found a significantly high prevalence of PI (73%), stress (44%), and current smoking (43%) among corporate workers in South Africa. Also, the prevalence of obesity (36%) and current alcohol consumption (29%) was moderately high among the South African workforce. However, the prevalence of hypertension is unexplainably low (9 %). It is also instructive to note that 21% of the South African workforce is already either prediabetic or diabetic, while more than half (55%) have already developed metabolic syndrome.

These results are also significantly higher than the WHO’s estimated prevalence of PI (37%), obesity (27%), and current tobacco use (20.3%) in South Africa [134,151]. This result is also far higher than Gallup’s estimated stress prevalence of 32% among South African workers [127]. However, this result is similar to the WHO’s estimated current alcohol consumption of 32.5% in South Africa [126]. Thus, the results of this study suggest that the prevalence of many CV risk factors is higher among the corporate workforce in South Africa than among the general population in the country, while cultural influences might have played a role in alcohol consumption among the South African corporate workforce.

In Nigeria, there was significantly high prevalence of unhealthy diet (79%), poor sleep (79%), stress (71%), and PI (62%) among the corporate workforce in this study. This result is lower than that of another study conducted among working-class Nigerian adults living in urban areas, where 80% of the participants did not meet the WHO’s minimal recommendations for physical activity [152]. However, the result is higher than Gallup’s estimate of 54% prevalence of stress among the Nigerian workforce [127]. This result is also significantly higher than the WHO estimate of 25% PI prevalence among Nigerians [151]. Thus, the findings of this study suggest that the prevalence of many CV risk factors is higher in the Nigerian workforce than in the general population in the country.

In Ethiopia, none of the corporate workers met the minimum requirement for a healthy diet. There was also a significantly higher prevalence of poor sleep (69%), stress (49%), PI (45%), and central obesity (40%). The different components of dyslipidemia were also significantly elevated (high LDL-C, 45%; low HDL-C, 55%; hypertriglyceridemia, 47%; and high TC, 26%). Furthermore, the prevalence of hypertension (30%), current alcohol consumption (34%), and high salt intake (23%) was moderately high. However, only 10% of the Ethiopian corporate workforce were obese, while 6% each were either currently smoking tobacco products or chewing khat.

These results are significantly higher than the WHO estimates of 14%, 6%, and 4% for PI, high salt intake, and obesity, respectively, among the general population in Ethiopia [150]. It is also slightly higher than the WHO’s estimated prevalence of current tobacco smoking (4.6%) and alcohol consumption (20%) among the general Ethiopian population [127,134].

In general, the results of this systematic review suggest that the prevalence of most CV risk factors is higher among the corporate workforce of each identified country in SSA than among the general population in these countries. This finding also supports the position that the unhealthy workplace culture and environment of many corporate organizations in SSA may contribute to the increasing prevalence of CVD risk factors in the region. In addition, there are significant variations in the distribution pattern of the risk factors across different countries, which is most likely due to cultural preferences in each country.

Thus, to significantly mitigate the rising prevalence of CVD and CV risk factors in SSA, it may be expedient that the government and corporate organization in each country enact policies and programs that effectively address these CV risk factors in the workplace. However, to be successful, such interventions must factor in the peculiar distribution of these CV risk factors and their cultural influences across different countries. Consequently, the International Labour Organization, WHO, and other stakeholders have suggested that all business organizations or workplaces in different countries should implement a robust workplace wellness program among their employees [15,153,154]. Such intervention may provide adequate awareness/education about CV risk factors to the workers and implement practical solutions to mitigate the modifiable risk factors.

Prevalence of Cardiovascular Risk Factors among the Corporate Workforce across Work Sectors in Sub-Saharan Africa

A systematic review revealed that 17 different CV risk factors were identified in the healthcare sector, making the sector have the highest cluster of CV risk factors. The healthcare sector is closely followed by the education sector (14), administration (10), finance (9), agro-allied (7), judiciary (5), manufacturing (4), and telecommunication with three identified CV risk factors. The most prevalent CV risk factors among healthcare workers were unhealthy diet (80%), poor sleep (61%), PI (53%), central obesity (51%), and stress (51%).

Also, the prevalence of central obesity, dyslipidemia, and hypertension (previous history of hypertension and hypertension on measurement) was 51%, 51%, and 41%, respectively. Conversely, the prevalence of dysglycemia and current tobacco smoking was only 11% and 7% respectively among healthcare workers. It is instructive to note that almost all the CV risk factors were significantly high among healthcare workers, with most identified risk factors recording more than 50%.

When compared to the other sectors, the healthcare sector had the highest prevalence of unhealthy diets, central obesity, and high TC. The healthcare sector also had the second highest prevalence of stress, history of hypertension, and high LDL-c. All these findings suggest that the high cluster and prevalence of most CVD risk factors among healthcare workers in SSA places the workers in this sector at a high risk of developing CVD, which is higher than many of the other sectors.

Healthcare workers are often expected to be most informed about CVD and CV risk factors. They are also expected to implement additional preventive measures. Therefore, it is puzzling to discover that the healthcare sector in SSA had the highest cluster of CV risk factors and a high prevalence of most individual CV risk factors. It is not impossible that this discovery was due to surveillance (detection) bias [153]. Compared to other work sectors, healthcare workers may conduct more screening tests among themselves because of their better knowledge about CVD and more accessibility to screening tools.

The poor healthcare system in SSA and unhealthy working conditions in many healthcare facilities in the region may also be contributing factors. Some of the root causes that have been suggested to contribute directly or indirectly to the poor healthcare system and unhealthy working conditions in the healthcare sector in Africa include poor leadership and management, insufficient and poorly trained human resources, uneven distribution of the workforce (urban versus rural distribution), inadequate budgetary allocation, heavy workloads and schedules, inadequate protection from occupational hazards, deteriorating medical infrastructure, and poor financial compensations [154-157]. To significantly reduce the prevalence of CVD risk factors among healthcare workers in SSA, it is expedient that all these root causes are comprehensively addressed, in addition to the provision of robust workplace wellness policies and programs for healthcare workers.

In the education sector, the most prevalent risk factors are - PI (75%), no fruit consumption (79%), stress (50%), and low HDL-c (51%). However, just like the healthcare sector the estimated prevalence of current smoking was low (7%) among the educators. When compared to the other work sectors, the education sector ranked highest in the prevalence of PI, second in no fruit consumption and in the cluster of individual CV risk factors, and third in both stress and central obesity. This result is supported by the findings of another study that noted that a significantly high percentage of academic staff in Nigeria do not engage in adequate physical activity [158].

It is not completely clear why PI or a sedentary lifestyle is the highest among education sector workers in SSA, or why they had the second highest cluster of CV risk factors. This may be because, in addition to unhealthy workplace culture, many education sector workers, especially lecturers and researchers, spend a significant amount of time behind the desktop/table to conduct research and other academic activities, with little time to engage in some healthy lifestyle practices such as adequate physical activity and sleep duration.

In the administration sector, the prevalence of unhealthy diet (75%), PI (68%), and central obesity (50%) are estimated to be significantly high compared to other risk factors in the sector. When compared with other sectors, the administration sector ranked highest in current tobacco smoking and dysglycemia; second in PI, obesity, and central obesity; and third in the cluster of CV risk factors. The comparatively high prevalence of PI, high BMI, and central obesity is particularly instructive. This may be because many modern administrative work schedules often demand working for long hours, seated behind a table or desktop, with little time to exercise.

Conversely, among those working in the finance, manufacturing, and agricultural sectors, the estimated prevalence of alcohol consumption was high, at 54%, 35%, and 44%, respectively. Making these sectors the highest current alcohol-consuming sectors in the study. Also, the prevalence of stress (62%) was highest among the finance sector workers, while the manufacturing sector chewed khat (8%) the most. In addition, PI was also estimated to be high in the finance (59%) and agro-allied (57%) sectors.

Some of the reasons that may make many bankers and other financial sector workers in SSA have a high prevalence of stress include high job demand, expected low margin of error, inflexible and heavy workloads, long working hours, poor working equipment and conditions, and frequent misunderstanding and conflicts with customers. Frequent alcohol consumption may be a negative stress coping strategy among financial workers.

The judiciary sector had the highest prevalence of overweight (52%) and obesity (45%) and the second highest prevalence of hypertension (40%) compared to other sectors. Almost half (45%) of the workers in the telecommunication section were overweight, 25% were obese, and 30% were hypertensive. The high prevalence of overweight, obesity, and hypertension among judicial staff may be due to the poor justice system (with inadequate funding, perceived corruption, and judicial bias) and slow judicial reform in many countries in Africa [159-162]. A poor justice system often results in a long judicial process which often translates to judicial workers sitting for a long period of time, resolving many complicated cases. This unhealthy working system may also increase the risk of developing hypertension by some of the judicial workers

The systematic review of the studies that investigated workers from different sectors (mixed sector) provided a summary of the prevalence of the different CV risk factors among the sub-Saharan workforce. In the mixed sector, there was a high prevalence of unhealthy diet (70%), PI (58%), and stress (48%). Many components of dyslipidemia were also high (low HDL was 61%, high LDL-c was 57%, hypertriglyceridemia was 52%, and high TC was 30%). Furthermore, overweight (31%), obesity (39%), central obesity (28%), and hypertension (32%) were moderately high among the mixed-sector workers. All of these moderate to very high prevalence of CV risk factors must have resulted in more than half (53%) of the workers in the mixed sector being classified as having metabolic syndrome [163-165].

All these results suggest that all the analyzed work sectors have a moderate to very high prevalence of most traditional CV risk factors. In addition, there is significant variation in the distribution patterns of most CV risk factors across different work sectors. Although all the sectors had peculiarities, the unhealthy workplace systems, culture, and environment in many of these work sectors may contribute to the high prevalence of CV risk factors among their staff. Many work sectors in Africa lack evidence-based workplace wellness solutions [144]. Thus, to reduce the risk of CVD among the corporate workforce in SSA, it may be expedient for all the work sectors to comprehensively review their peculiar unhealthy workplace challenges and provide evidence-based and sector-specific solutions. There are numerous studies that suggest that well-designed and delivered workplace wellness programs can significantly mitigate the modifiable CV risk factors among corporate workers and further research in this direction will be crucial [166-168].

Limitations

There were inconsistent screening and diagnostic criteria for some of the identified risk factors (e.g., hypertension, unhealthy diet, and poor sleep) across the studies. Also, some of the studies involved different cadre of participants from different work sectors.

## Conclusions

The result of the study revealed moderate to high prevalence of most of the CV risk factors in all the regions, countries, and work sectors in SSA. The prevalence of most of these risk factors is higher in the corporate workforce in most regions and countries in SSA compared to the general population in the region. Consequently, compared to the general population, without deliberate mitigating actions, the corporate workforce in SSA may experience a high prevalence of CVD in the near future. A situation that may result in a slower or even a decline in the economic growth of the region. Also, there is a significant variation in the distribution of most CVD risk factors across the regions, countries, and work sectors, most of which may be culturally influenced. Future studies should investigate the exact reasons for the variations in the distribution of CVD risk factors across regions, countries, and work sectors.

To mitigates the rising CVD epidemics in SSA, it may be necessary for the government and employers in different regions, countries and work sectors in SSA to consider mass health awareness/education programs on the rising prevalence of CVD and its risk factors in SSA, especially among the corporate workforce in the region. Also, there is a need to deploy more resources for screening of CVD risk factors among the workers. In addition, evidence-based workplace wellness solutions (policy and programs) that identify and mitigate the major CV risk factors need to be deployed in all the countries and work sectors in SSA.

However, considering the peculiar regional distribution of these risk factors and possible cultural influences, workplace wellness solutions should be personalized to each region, country, and work sector. A personalized workplace wellness solution may include organizing regular stress management programs for finance workers, providing physical activity incentives for educators, or subsidizing gym membership for healthcare workers. Lastly, there is a need to standardize the diagnostic criteria for some of the CV risk factors, especially the behavioral risk factors. Also, further research is needed to identify the causal mechanisms for the high prevalence of most CV risk factors among the workforce in SSA.

## Appendices

Operational definition of terms

Behavioral risk factors: These are behaviors, lifestyles, or habits that exert a strong negative influence on health and significantly increase the risk of developing CVD.

Intermediate risk factors: These are biophysical or biochemical bodily changes, many times in response to prolonged exposure to behavioral risk factors, that increase the risk of developing CVD.

Cluster of cardiovascular risk factors: These are groups or aggregate of different cardiovascular risk factors occurring in the same work sector in sub-Saharan Africa.

Corporate workforce/workers: These are people who work or perform professional managerial or administrative services usually behind the desk or in a formal workplace.

Current tobacco use: Use of any tobacco-containing products (such as cigarettes, cigars, pipe, leaf, snuff, e-cigarettes, etc.) in the last 1 month.

Harmful alcohol consumption: Intake of more than 2 drinks of alcohol daily for men or more than 1 drink daily for women; or intake of 5 or more alcohol bottles on an occasion for men or 4 or more alcohol bottles on an occasion for women; or any risky or unsafe alcohol intake as determined by a validated alcohol use screening tool (such as the Alcohol Use Disorders Identification Test - AUDIT; Cut, Annoyed, Guilty, and Eye - CAGE, etc.).

Physical inactivity: Getting less than 30 minutes/day or 150 minutes/week of moderate-intensity aerobic physical activity, or less than 20 minutes/day or 75 minutes/week of vigorous-intensity aerobic physical activity, or physical inactivity as measured by a validated physical activity screening tool (such as the Global Physical Activity Questionnaire - GPAQ, International Physical Activity Questionnaire - IPAQ, etc.).

Poor Sleep: Sleeping less than 7 hours most nights (Dedhia and Maurer, 2022), or poor sleep quality as measured by a validated sleep quality screening tool (such as the Pittsburgh Sleep Quality Index - PSQI).

Stress: Stress is the physical and mental response to a perceived or real threat. It is measured by a validated stress screening tool (Such as the Depression, Anxiety, and Stress Scale - DASS), Perceived Stress Scale - PSS, etc.).

Unhealthy diets: Consumption of fewer than 5 servings of fruits and/or vegetables in a day; and/or regular consumption of foods high in sugars, salt, and/or fats, or any diet that is categorized as unhealthy by a validated healthy diet screening tool (such as the Mediterranean Diet Adherence Screener - MEDAS).
